# Targeting chaperonin containing TCP1 (CCT) as a molecular therapeutic for small cell lung cancer

**DOI:** 10.18632/oncotarget.22681

**Published:** 2017-11-25

**Authors:** Ana C. Carr, Amr S. Khaled, Rania Bassiouni, Orielyz Flores, Daniel Nierenberg, Hammad Bhatti, Priya Vishnubhotla, J. Perez Manuel, Santimukul Santra, Annette R. Khaled

**Affiliations:** ^1^ Burnett School of Biomedical Science, College of Medicine, University of Central Florida, Orlando, FL 32827, USA; ^2^ Department of Pathology and Laboratory Medicine, Department of Internal Medicine, Orlando VA Medical Center, Orlando, FL 32803, USA; ^3^ Biomedical Imaging Research Institute, & Samuel Oschin Comprehensive Cancer Institute, Department of Biomedical Sciences and Department of Neurosurgery, Cedar Sinai Medical Center, Los Angeles, CA 90048, USA; ^4^ Department of Chemistry, Pittsburg State University, Pittsburg, KS 66762, USA

**Keywords:** chaperone, lung cancer, peptide, STAT3, nanoparticles

## Abstract

Identifying new druggable targets is desired to meet the needs for effective cancer treatments. To this end, we previously reported the efficacy of a therapeutic peptide called CT20p that displays selective cytotoxicity through inhibition of a multi-subunit, protein-folding complex called Chaperonin-Containing TCP-1 (CCT). To investigate the role of CCT in cancer progression, we examined protein levels of CCT subunits in liver, prostate, and lung cancer using human tissue microarrays. We found that these cancers expressed higher levels of CCT2 as compared to normal tissues. Small cell lung cancer (SCLC) stood out as having statistically significant difference in CCT2. Higher levels of CCT2 in tumors from lung cancer patients were also associated with decreased survival. Using SCLC cell lines, we observed detectable amounts of CCT subunits and cells were susceptible to killing by CT20p. Treatment with CT20p, delivered to cells using polymeric nanoparticles, was cytotoxic to all SCLC cell lines, decreasing the levels of CCT client proteins like STAT3. In contrast, treatment with a STAT3 inhibitor was effective in one of the SCLC cell lines. While we found that CCT levels could vary in cell lines, normal tissues had low levels of CCT and minimal toxicity to liver or kidney function was observed in mice treated with CT20p. These results indicate that in SCLC, changes in CCT levels could be used as a biomarker for diagnosis and that targeting CCT for inhibition with CT20p is a promising treatment approach for those cancers such as SCLC that currently lack targeted therapeutics.

## INTRODUCTION

Cancer remains a leading cause of death, with breast, lung, prostate, and colorectal among the top four cancers in terms of new cases diagnosed and estimated deaths [1]. Most cancer-related deaths are due to metastatic disease, when the primary cancer spreads to vital organs like the brain, bone, or liver. A significant impediment to finding effective treatments or better ways to detect and diagnose cancers, especially metastatic cancers, is tumor heterogeneity. Genomic instability, together with selection pressures from the tumor micro-environment, are among the factors that produce genetic and phenotypic differences in patients’ tumors and even within the same tumor (intra-tumor heterogeneity). Case in point is small cell lung cancer (SCLC). SCLC accounts for about 15% of lung cancer cases and is characterized by genetic alterations and a high proliferative index as well as early metastatic spread [2]. SCLC is an aggressive disease, and while responsive to initial treatments, patients later succumb to the disease, which is associated with a high rate of relapse and recurrence [3, 4]. SCLC can be further classified as limited stage (LS-SCLC) if the disease is confined to one hemithorax and regional nodes, or as extensive disease (ES-SCLC) if the cancer has spread to distant sites [5, 6]. This classification helps determine the treatment plan for SCLC patients, whether surgery and/or prophylactic cranial irradiation (PCI) are recommended. The first-line treatment for any stage of SCLC is etoposide-platinum based chemotherapy; however, patient survival decreases upon disease recurrence, largely due to the development of chemoresistance [3, 7, 8]. Although SCLC is a good candidate for targeted therapies, clinical trials testing new targeted chemotherapeutics for SCLC patients have been unsuccessful. As an example, a recent clinical trial using bevacizumab—an angiogenesis inhibitor targeting the vascular endothelial growth factor (VEGF)—in combination with etoposide-platinum based chemotherapy for SCLC patients, concluded that the addition of bevacizumab did not improve patient overall survival [9]. In comparison, in non-small cell lung cancer (NSCLC) patients, modest results were achieved with angiogenesis inhibitors like bevacizumab when used in combination with chemotherapeutics to treat metastatic patients [10, 11]. While there is renewed hope of developing new cancer drugs, especially with the recent focus on immunotherapies, the challenges of achieving a molecularly targeted treatment that provides real benefit for patients with cancers like SCLC are substantial. Central to these challenges is the need to identify druggable molecular alterations through a better understanding of the basic biology underlying these aggressive cancers.

The molecular landscape underlying the heterogeneity of cancers like SCLC is far from understood and complicates cancer drug discovery. While SCLC has not undergone extensive molecular subtyping, it is characterized by a high mutation rate and genomic instability. Inactivation of TP53 and RB1 [12], along with alterations of MYC, NOTCH, and the PI3K/AKT/mTOR pathways, are among the key genetic changes associated with SCLC [13]. Other genes less frequently mutated in SCLC include *EGFR* [14], *KIT* [15], *MET* [16, 17], and heat shock proteins [18]. These genes are also commonly mutated in other cancers. [19]. Using targeted agents to inhibit these pathways is the goal of many studies, but recent findings suggest that single target agents are less effective, and that combination therapies, hitting multiple pathways, are more successful in preventing cancer relapse and drug resistance in patients [20, 21]. A drawback for combination approaches, however, is the potential for increased drug-related off-site toxicities. A rational approach would be to inhibit protein-folding, which could impact multiple pathways while using a single inhibitory agent. Chaperonin-containing TCP-1 (CCT) or T-complex 1 ring protein (TRiC) is an evolutionarily conserved macromolecular complex involved in folding about 10% of the cell proteome [22]. Many of the gene products deregulated in cancers such as SCLC (e.g., *MYC*, *TP53*, *CCNE, KRAS, HSP90, ESR1*, and *NOTCH*) are CCT client proteins [23], suggesting a role for the involvement of CCT in the development of cancer, driving the need to better understand its contribution to malignant transformation.

Collective evidence suggests that CCT may be upregulated in a broad range of cancers. CCT is a hetero-oligomeric complex composed of two rings formed by eight subunits [24]. Each CCT subunit, named CCT1-8 (or CCTα-θ), is found at a fixed position in the ring [22]. Protein folding occurs within a central chamber formed by the rings and is mediated through the opening and closing of a lid structure in an ATP-dependent fashion [25, 26]. A study examining CCT and its activity in cancer cell lines revealed that while these cell lines had varying reliance on the protein-folding activity of CCT, it was more impactful in cancer cells than in normal cells [27]. As an example, in hepatocellular (HCC) carcinoma, the CCT8 subunit was upregulated and played an important role in the proliferation of these cells [28]. A similar correlation was observed with CCT1 (TCP1) and CCT2 in both hepatocellular carcinoma and colonic carcinoma [29], as well as with CCT2 in colorectal carcinoma. In breast cancer, CCT1 and CCT2 may be upregulated by driver oncogenes that are responsible for tumorigenesis [30]. Our own published studies provided evidence that CCT2 is significantly overexpressed in invasive ductal breast carcinoma and in triple-negative breast cancer (TNBC) cell lines, like MDA-MB-231 [31], as well as prostate cancer cell lines [32]. This suggests that the CCT2 levels could correspond to increased disease severity.

The elevated expression of different CCT subunits in cancer is intriguing on two fronts. First, CCT levels may be useful as a biomarker in clinical settings. Potentially, CCT could be detected as an early diagnostic marker in cancers such as SCLC. Or it could be used for the diagnosis of disease severity in cancers like breast cancer, in which levels of CCT2 correlated with disease progression, as we have previously demonstrated [31]. Second, CCT is a promising target for cancer therapy, as depletion of CCT in cancer cells caused cell cycle arrest and growth inhibition [28, 30]. In previous studies, we showed that an amphipathic peptide called CT20p, derived from the last twenty amino acids of Bax, had cytotoxicity activity that was independent of the parent protein or Bcl-2 overexpression and caused cytoskeletal disorganization, loss of adhesion, and cell death [33, 34]. CT20p, while being membrane impermeable, can be readily encapsulated for delivery to cancer cells in nanoparticles (NPs) made from a hyperbranched, polyester polymer [35, 36]. In animal models of breast and prostate cancer, treatment with CT20p-NPs caused significant tumor regression [31, 32]. The mechanism of action of CT20p is likely inhibition of CCT, as overexpression or deletion of the CCT2 subunit altered the capacity of the peptide to kill cells [31]. In the work presented herein, we examined the protein levels of CCT in tissue samples of liver, prostate, colon, and lung cancer to determine whether CCT subunits were highly expressed in these cancers. We also sought to determine whether inhibition of CCT through treatment with CT20p could impact CCT client proteins needed for cancer progression. To that end, we focused on STAT3, since this transcription factor is a CCT client protein [37], is constitutively active in many tumors [38], and is a predictor of poor prognosis in cancer [38, 39]. Our analysis revealed SCLC to be a promising model to study the role of CCT in cancer, as well as a likely candidate to benefit from CCT-targeted therapy.

## RESULTS

### CCT2 is overexpressed in lung cancer patient tumors and correlates with decreased survival

**Figure 1:**
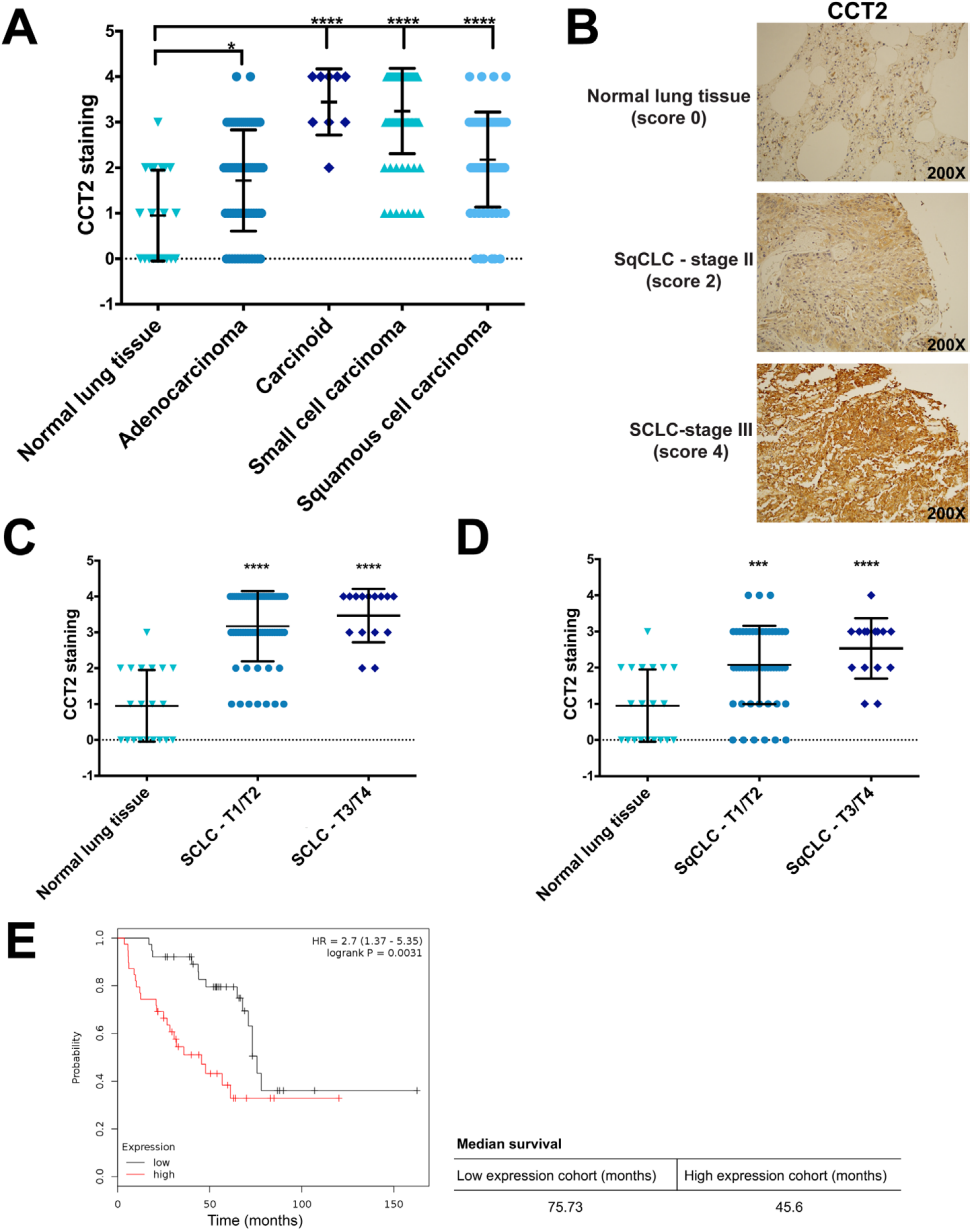
Analysis of CCT2 staining in lung cancer patient tissue **(A)** The levels of CCT2 were assayed in human tumor tissue samples of different lung cancer subtypes by immunohistochemistry (IHC) as described in Materials and Methods. Tissue cores were analyzed by a pathologist according to stain intensity and given a score between 0-4 as previously published in Bassiouni et al (2016). Significance was calculated in reference to normal lung tissue. Number of samples used for the analysis can be found in Supplementary Table 1. **(B)** Representative images of stained human tissue microarrays (TMAs) shows the elevated levels of CCT2 in both squamous cell carcinoma and small cell carcinoma compared to low levels in normal lung tissue. **(C)** Small cell lung cancer (SCLC) samples were grouped according to TNM score (T1/T2 and T3/T4) and stain intensity for CCT2 was compared between them. Significance indicated is in reference to normal lung tissue. **(D)** Squamous cell lung carcinoma (SqCLC) was also grouped by TNM score (T1/T2 and T3/T4) and CCT2 stain intensity was compared. Significance indicated is in reference to normal lung tissue. **(E)** Kaplan-Meier plot of lung cancer patients based on CCT2 expression. The survival graph was generated using publicly available database (KM plotter; www.kmplot.com). Data analysis was restricted to patients with grade III lung cancer (n=77). ^*^ = p<0.05, ^**^ = p<0.01, ^***^ = p<0.001, ^****^ = p<0.0001.

CCT is a macromolecular complex composed of eight subunits, which we will refer to by number (CCT1-8) hereinafter. Our previous studies with breast cancer [31] revealed that the CCT2 subunit was overexpressed in tumor tissues as compared to normal tissues, was increased with advanced disease, and was inversely correlated with patient survival. To determine whether CCT2 was elevated in other cancers, we evaluated CCT2 protein levels in several human tissue microarrays (TMAs) for lung, colon, hepatocellular, and prostate carcinomas by immunohistochemistry (IHC). We found that lung, liver, and prostate tissues had significantly higher levels of CCT2 as compared to matched normal tissue (Figure 1A, 1B, Supplementary Figure 1A, 1B and 1E, 1F and Supplementary Tables 1 and 2). Our findings for colon cancer were inconclusive due to high background staining of normal colon tissue (Supplementary Figure 1). In liver carcinomas, CCT2 levels were higher in both subtypes analyzed (HCC and cholangiocellular carcinomas) as compared to normal hepatic tissue (Supplementary Figure 1A). This difference was statistically significant (p<0.05) in HCC. We also analyzed HCC according to grade, as high grade HCC is associated with poorer prognosis [40], and observed a progressive increase in CCT2 staining with increasing grade (Supplementary Figure 1A). In prostate adenocarcinoma, we observed significantly increased levels of CCT2 as compared to normal prostate tissue (Supplementary Figure 1E). Because increased stage indicates higher severity of disease and poorer prognosis, we also analyzed CCT2 levels in prostate cancer by stage and observed a trend of increasing CCT2 staining with increasing stage (Supplementary Figure 1E). Mining databases like The Cancer Genome Atlas (TCGA) and the Human Protein Atlas confirmed our findings that high expression of CCT2 occurs in liver and prostate cancer and is associated with unfavorable prognosis (Supplementary Figure 1C, 1D and 1G, 1H) but in colon cancer, high levels of CCT2 were associated with improved prognosis, although this result was not statistically significant (Supplementary Figure 1J).

We next assessed the levels of CCT2 in various subtypes of lung cancer by evaluating staining intensity in 236 tissue microarray cores containing cases of adenocarcinoma, carcinoid, SCLC, and squamous cell lung carcinoma (SqCLC) (Supplementary Table 1). This was then compared to normal lung tissue. All lung cancer subtypes examined had significantly higher levels of CCT2 as compared to normal tissue (Figure 1A). Representative images of CCT2 staining in normal lung tissue, SqCLC, and SCLC are provided in Figure 1B. We selected SqCLC and SCLC to study in greater detail because together, these two lung cancer types represent 35%-45% of all cases [41] and both scored significantly higher than normal tissue for CCT2 (Figure 1A). To determine if CCT2 levels increased with disease progression in these two lung cancer subtypes, we grouped TMA cores according to their TNM classification (Figure 1C, 1D) or stage (data not shown) and found no statistically significant differences among the groups analyzed. Essentially, CCT2 levels were higher than normal lung in these lung cancer subtypes and independent of stage or grade. To investigate if CCT2 levels correlated with survival in lung cancer patients, we used the publicly available Kaplan-Meier plotter database to generate survival data in all lung cancer patients that expressed high levels of CCT2 mRNA. We found that there was a statistically significant correlation (p = 0.0031) between decreased survival of grade III lung cancer patients and CCT2 expression, with a 30-month difference in survival between the higher expression patient cohort and the lower expression patient cohort (Figure 1E). These results were confirmed with the TCGA database (Supplementary Figure 2).

We and others previously reported a correlation between high levels of CCT subunits and disease progression in breast [31], hepatocellular and colonic carcinoma, and gallbladder squamous/adenosquamous carcinoma [42]. To our knowledge, however, no other groups have published a correlation between lung cancer and CCT levels. To address this gap, we selected this cancer type to investigate further.

### SCLC cell lines express varied levels of CCT2, CCT4, and CCT5 subunits and were susceptible to killing by CT20p

**Figure 2:**
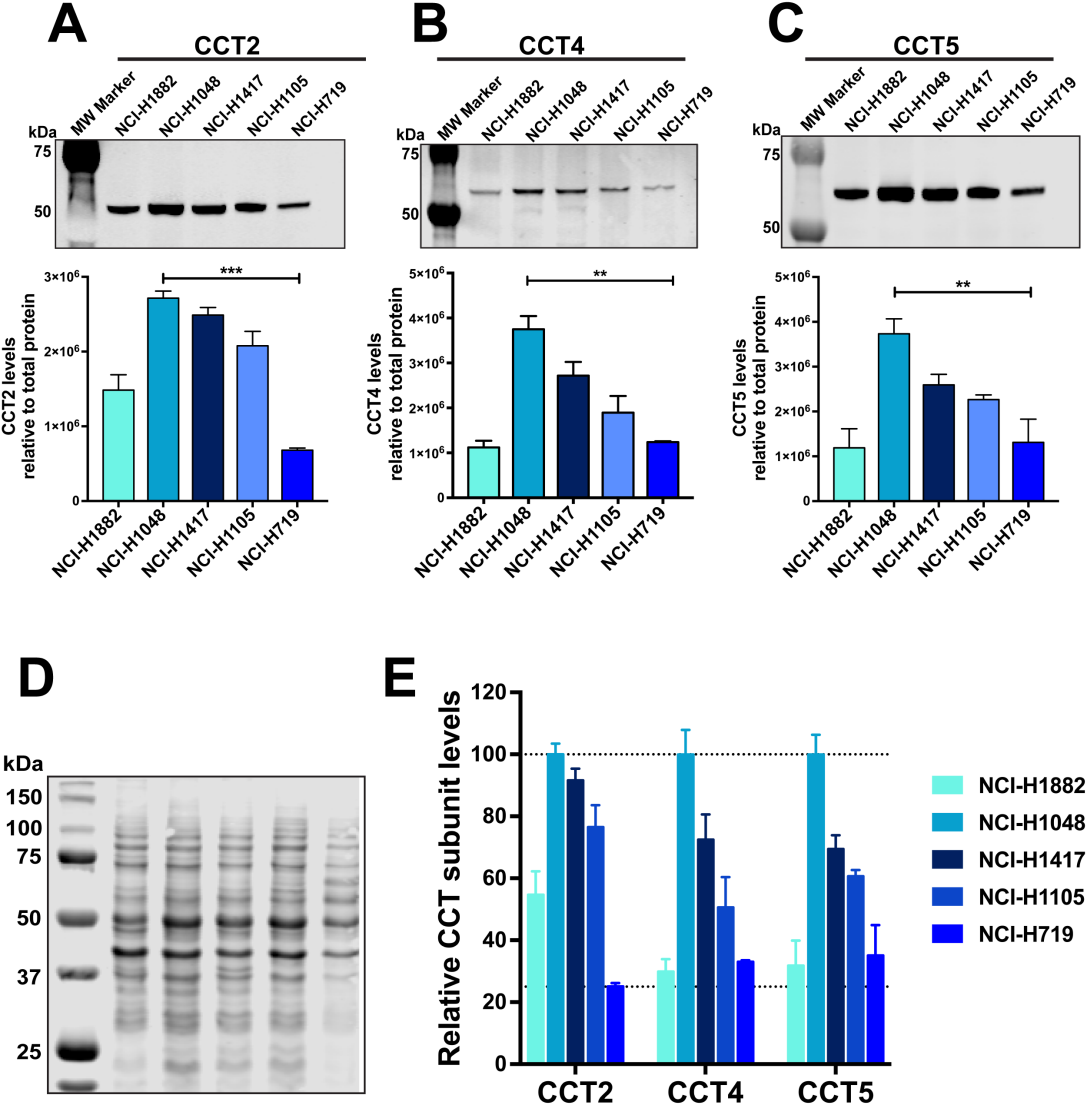
Levels of CCT subunits are detected in SCLC cell lines Lysates corresponding to five SCLC cell lines were subject to immunoblot analysis for **(A)** CCT2, **(B)** CCT4 and **(C)** CCT5. The bar graphs below each corresponding blot, shows their relative levels for all five cell lines. Relative quantification was calculated by normalizing antibody specific signal to signal obtained from total protein. **(D)** PVDF membrane stained for total protein using Revert (LI-COR). **(E)** Graph showing relative values of the three assayed CCT subunits in each SCLC cell line. ^*^ = p<0.05, ^**^ = p<0.01, ^***^ = p<0.001, ^****^ = p<0.0001. Blot images are representative.

About 95% of SCLC patients have TP53 mutations [43]; therefore, as representative cell lines for our studies, we chose four SCLC cell lines containing common TP53 mutations and one SCLC cell line with wild-type TP53 (Supplementary Table 3). To assess CCT expression in the SCLC cell lines, we determined the basal protein levels of the subunits CCT2, CCT4, and CCT5 by immunoblot (Figure 2A-2C). The levels of assayed subunits were normalized to total protein by staining the membrane prior to blocking. Figure 2D provides a representative image of the membrane stained for total protein. The cell line NCI-H1048 had the highest level of all five cell lines assayed for CCT subunits (Figure 2A-2C). In contrast, NCI-H719 had the lowest level of CCT2, CCT4, and CCT5 (Figure 2A-2C). The levels of CCT subunits in cell lines NCI-H1882, NCI-H1417, and NCI-H1105 were in between those of the highest and lowest SCLC cell lines (Figure 2A-2C). For a qualitative analysis of relative levels of the assayed CCT subunits, we normalized the signal from all cell lines to the signal from cell line NCI-H1048 for each CCT subunit assayed. We then combined the data into one graph to aid visualization (Figure 2E). It is important to note that this comparison is only qualitative since different antibodies were used to detect each subunit. The relevant knowledge we gained from these experiments is that we can detect protein level differences of each CCT subunit among the selected cell lines. As a quality control check for our protein lysates, we also assayed basal levels of wild-type (WT) or mutant p53 in these cell lines and found that NCI-H719 cells had the highest and NCI-H1417 cells had the lowest levels of mutant p53, which confirmed the data set available at the ATCC website (Supplementary Figure 3).

**Figure 3:**
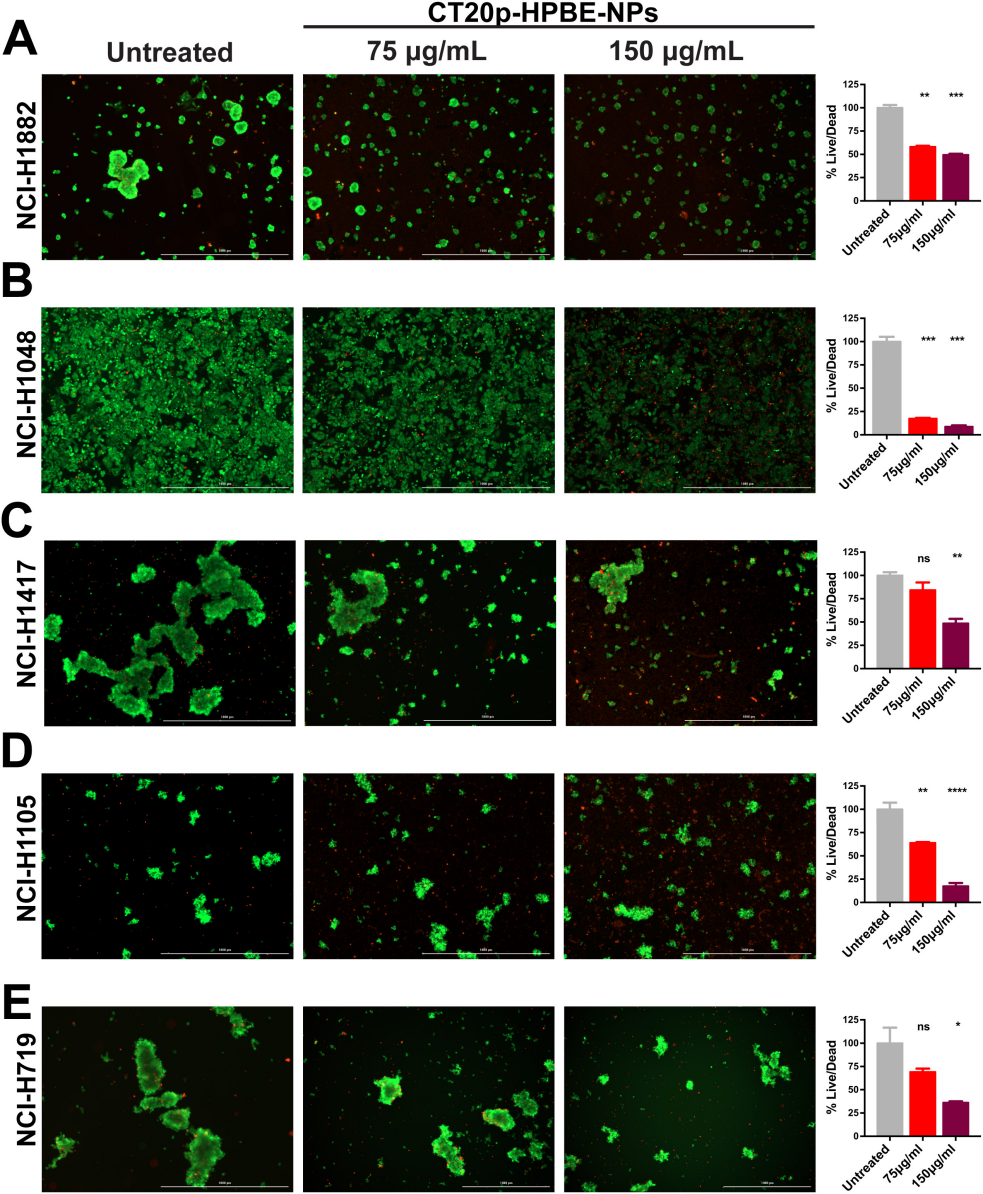
CT20p-NPs is cytotoxic to SCLC cell lines Five SCLC cell lines were subject to CT20p treatment at 75 μg/ml or 150 μg/ml for 48 hrs as described in Methods and Materials. Untreated control received medium only. Calcein-AM and ethidium homodimer were added to cell cultures prior imaging to indicate live (green) and dead (red) cells respectively. Representative images of **(A)** NCI-H1882, **(B)** NCI-H1048, **(C)** NCI-H1417, **(D)** NCI-H1105 and **(E)** NCI-H719 are shown for untreated control and the two doses used. Signal obtained using GFP filter (indicative of live cells) was divided by signal detected using TexasRed filter (indicative of dead or damaged cells) for each cell line. Bar graphs adjacent to each corresponding cell line show the normalized ratios (%live/dead). Cytotoxic effects can be observed by an overall decrease in green signal concomitantly to an increase in red signal. ^*^ = p<0.05, ^**^ = p<0.01, ^***^ = p<0.001, ^****^ = p<0.0001.

Our previous published work showed that CT20p is selectively cytotoxic to breast cancer cells, and that modulating the levels of CCT2 by inhibition or overexpression regulated the susceptibility of cells to killing by CT20p [31]. Hence, it is likely that CT20p's primary mechanism of action is inhibition of CCT. To deliver CT20p to cells, we encapsulated the peptide in nanoparticles made from a hyperbranched polyester polymer (CT20p-NPs). These nanoparticles are approximately 80 nm in diameter, monodispersed and spherical as we have shown in our previous publications [31–34]. Peptide loading efficiency was estimated to be 0.15 μg CT20p per mg polymer. These nanoparticles are negatively charged and we confirmed uptake by all SCLC cells, using nanoparticles co-loaded with the fluorescent dye DiI (Supplementary Figure 4). All cells were treated with 75 and 150 μg/mL of CT20p-NPs (based on polymer amounts) for 48 hours and live/dead cells detected with two probes to assess intracellular esterase activity (Calcein AM) for live cells and plasma membrane integrity (ethidium homodimer) for dead cells. Live cells fluoresced green, while dead cells fluoresced red. The cell line most susceptible to CT20p treatment at the two doses tested was NCI-H1048 (Figure 3B), which also displayed the highest levels of all three CCT subunits (Figure 2A-2C). The cell line with the lowest amount of all three CCT subunits, NCI-H719, was also killed by CT20p treatment but to a lesser extent (Figure 3E). Interestingly, the only cell line with WT p53, NCI-H1882, had the second lowest levels of CCT subunits but was also highly susceptible to CT20p treatment (Figure 3A) This suggests that loss of p53, a CCT client protein, could contribute to cell death in this line. Hence, total levels of CCT may not always be as important as the outcome of CCT protein folding activity. The two cell lines with high CCT2 levels but lower levels of the other subunits, NCI-H1417 and NCI-H1105, were susceptible to CT20p, more so for NCI-H1105 (Figure 3C, 3D). These results indicate that while all SCLC cell lines were killed by CT20p-NPs, those like NCI-H1048 that upregulated multiple CCT subunits were more sensitive to killing by the peptide. We also observed that in a number of these cell lines, the cells tended to aggregate or cluster (NCI-H719, NCI-H1882), which also decreased upon treatment with CT20p (Figure 3E, 3A). This is important as we have shown that CCT inhibition through CT20p reduces actin and tubulin, which are cytoskeletal components that are CCT client proteins [31, 34]. Additionally, of the three SCLC cell lines with the highest levels of CCT subunits (NCI-H1048, NCI-H1417, and NCI-H1105), those derived from metastatic sources were more susceptible to killing by CT20p than those derived from a primary tumor source. These results are summarized in Supplementary Table 4.

### CCT2 levels correlate with STAT3 levels in SCLC patient tissues

**Figure 4:**
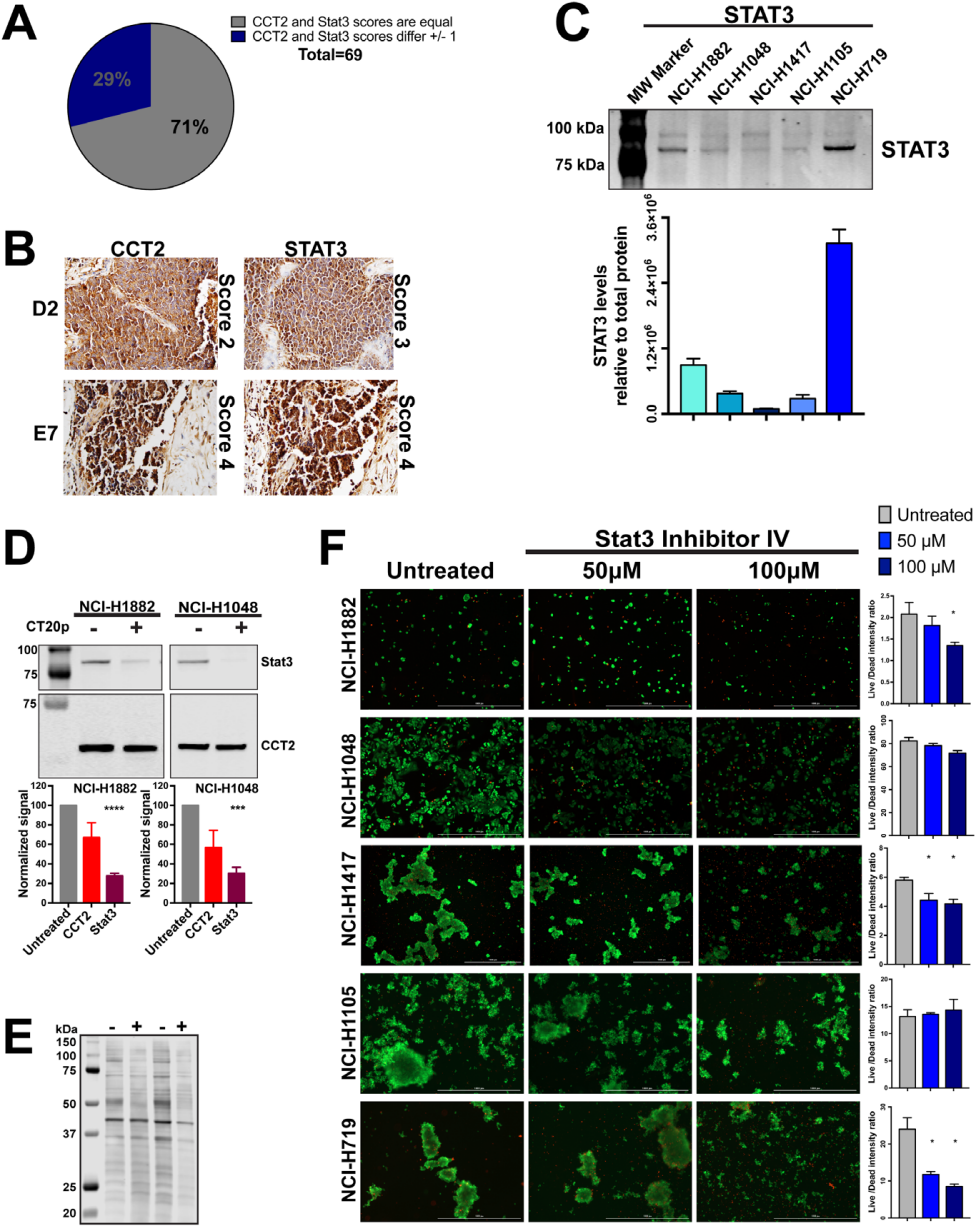
CT20p-NPs treatment decrease levels of client protein Stat3 in SCLC cell lines **(A-B)** TMA containing human SCLC tissue was stained for CCT2 and Stat3 in parallel as described in Methods and Materials. Number of samples and specific TMA information can be found in Supplementary Table 5. Tissue cores were analyzed by a pathologist according to stain intensity as described in Figure 1. (A) Pie chart indicates the number of cores which received equal scoring for CCT2 and Stat3 staining, as well as cores which received scores that differed by 1 (no cores received scores that differed by more than 1). (B) Representative images of corresponding CCT2 and Stat3 stained TMA cores D2 and E7 respectively. Images were provided by the pathologist at 200X (D2 pair) and 400X (E2 pair). **(C)** Immunoblot showing relative levels of Stat3 in the five SCLC cell lines. Levels of Stat3 shown were normalized to total protein as in Figure 2A-2C. Calculations are described in Methods and Materials. **(D)** Cell lines NCI-H1882 and NCI-H1048 were treated with 75 μg/ml CT20p-NPs for 16 hours and 6 hours respectively, at which point cells were trypsonized and lysates made. Soluble lysates of untreated cells (cultured in parallel with treated cells and lifted at the same time) were immunoblotted for CCT2 and Stat3. Bar graphs indicate relative levels of CCT2 and Stat3 in control and treated cells calculated as in Figure 2A-2C. **(E)** Image of stained PVDF membrane showing total protein corresponding to the samples blotted. Signal obtained from total protein was used to normalize CCT2 and Stat3 signal. **(F)** All five SCLC cell lines were treated with Stat3 Inhibitor IV at 50μM and 100μM. Untreated control received medium only at time of treatment. Live/Dead signal was detected and bar graphs generated as in Figure 3 and as described in Methods and Material. Representative images are shown. ^*^ = p<0.05, ^**^ = p<0.01, ^***^ = p<0.001, ^****^ = p<0.0001.

CCT is responsible for folding ∼5-10% of the cell's proteome [44] and many of its client proteins are involved in oncogenesis; however, the full CCT interactome remains to be discovered [23]. To determine whether CT20p treatment results in decreased CCT client proteins, we evaluated STAT3 because this transcription factor is frequently involved in oncogenesis [45] and its biosynthesis and activity is directly regulated by CCT [37]. To demonstrate a correlation between STAT3 and the levels of CCT2 in tumors from SCLC patients, we used TMAs containing SCLC tumor cores (Supplementary Table 5) for analysis of both CCT2 and STAT3. We found that the levels of STAT3 consistently correlated with CCT2 (Figure 4A) in most SCLC patient tumor tissues. Representative images of two STAT3/CCT2 pairs showing lower score (score 2 and 3) and high (score 4) are in Figure 4B. Next, we assayed total STAT3 protein levels in SCLC cell lines and determined that STAT3 was detectable in all five cell lines with the highest levels in NCI-H719 (Figure 4C). To investigate the effect of CT20p-NPs treatment on the levels of total STAT3, two adherent SCLC cell lines that were most sensitive to the peptide, NCI-H1882 and NCI-H1048, were subjected to treatment with the peptide for 16 hours and 6 hours respectively. These times were chosen in order to obtain enough viable cells for high quality cell lysates. Protein lysates were not effectively recovered from SCLC cells treated with CT20p-NPs beyond 16 hours, indicating that most cells after treatment are likely dead or dying. CCT2 and STAT3 protein levels were normalized to total protein to control for any proteolysis in the CT20p-treated cells. We found that in both SCLC cell lines, STAT3 levels decreased significantly, supporting the notion that CT20p could be inhibiting the protein-folding activity of CCT and causing loss of client proteins like STAT3 (Figure 4D). We also noted that the levels of CCT2 itself also decreased, but to a lesser extent. That CT20p could cause destabilization and cause the loss of CCT subunits is intriguing and indicates that more needs to be learned about the regulation and formation of the CCT complex.

We also treated the SCLC cell lines with a STAT3 inhibitor (STAT3 inhibitor VI) that binds to the SH2 domain and prevents STAT3 phosphorylation. Previous published studies showed a reduction in tumor cell growth *in vitro* at doses less than 100 μM [46, 47]. SCLC cells were treated for 48 hours at 50 and 100 μM and viability assessed as in Figure 3. We found that other than the SCLC line, NCI-H719, the SCLC cells were minimally affected by the STAT3 inhibitor (Figure 4F). This suggests that CT20p was more effective at reducing STAT3 levels and killing cells than a typical STAT3 inhibitor that targets only the phosphorylation and activation of the transcription factor.

### Immortalized and actively dividing cells have high levels of CCT2

**Figure 5:**
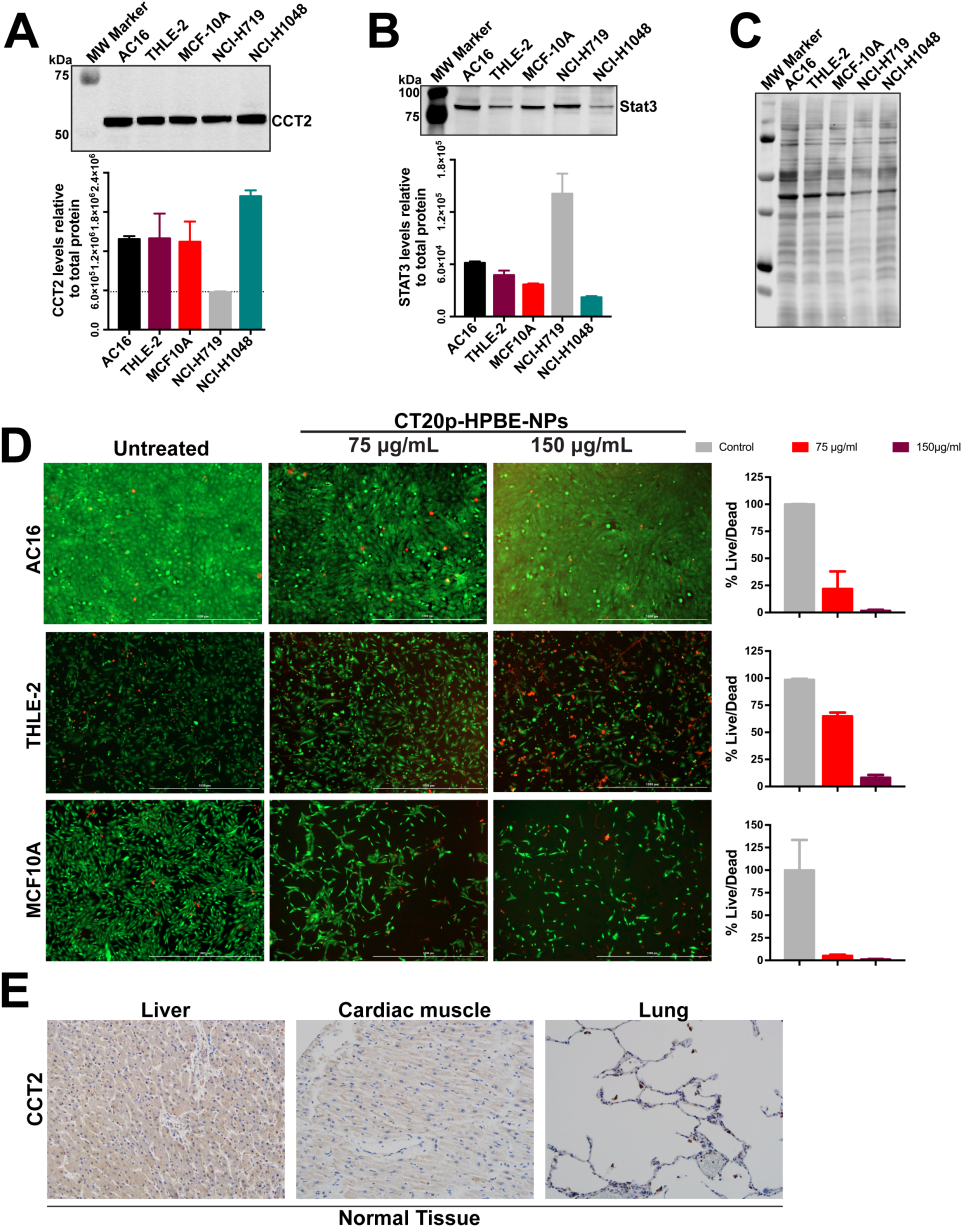
Immortalized or dividing cells but not normal tissues express CCT2 **(A-B)** Immunoblot of the indicated immortalized cell lines alongside two SCLC cell lines for CCT2 (A) and STAT3 (B). **(C)** PVDF membrane stained for total protein, used for normalization of band intensity after antibody probing. **(D)** Viability of immortalized cell lines after 48 hours treatments with CT20p-NPs at indicated concentrations and control was assessed using Calcein-AM and ethidium homodimer to indicate live and dead cells respectively as described in Figure 3. **(E)** The levels of CCT2 were assayed in tissue from normal organs by IHC as described in Materials and Methods. Representative images of stained TMAs of liver, heart and lung shows minimal levels of CCT2 detected.

To examine the broader effect of CT20p treatment on other cell lines, we selected three immortalized non-cancer cell lines that are typically used as controls for testing chemotherapeutics [48]. The AC16 and THLE-2 cell lines are used to examine cardio and liver toxicities. Based on our previously published work, the normal breast cell line MCF-10A was also selected. We first determined the basal levels of CCT2 (Figure 5A) and STAT3 (Figure 5B) and included two SCLC cell lines, NCI-H1048 and NCI-H719, for comparison since NCI-H1048 had the highest level of CCT2 and NCI-H719 had the lowest (Figure 2A). Note that Figure 5C contains a representative image of one of the blots used for relative quantification of the target levels as well as the total protein stain used for normalization. AC16 and THLE-2 cells had levels of CCT2 that were even higher than one of the SCLC cell lines, NCI-H719 (Figure 5A). This was expected since both AC16 and THLE-2 cell lines were immortalized using SV40 oncogene [49, 50]. SV40 inhibits p53 and Rb1, inactivation of which are hallmarks of SCLC. The conditional inactivation of these alleles in mice was sufficient for the mice to develop SCLC [51]. We also observed a similar trend of high CCT2 levels when cells like the immortalized lung cancer cell line, BEAS-2B (data not shown), and low passage, normal human bronchial epithelial (NHBE) or fibroblast cells (NHLF) (Supplementary Figure 5) were actively replicating in cell culture, likely due to the need for increased CCT client proteins like cyclins. Since it is known that NHBE cells can cause phenotypic changes when co-cultured with cancer cells [52] future studies will investigate CCT levels under co-culture conditions and in senescent or differentiated versus dividing cells to determine the involvement of the chaperonin in modulating the behavior of normal and cancer cells. In this regard, in our previous work [31], we found that MCF-10A cells can undergo spontaneous epithelial-to-mesenchymal (EMT) transformation and that transformed cells exhibit higher levels of CCT2 and are susceptible to CT20p treatment as shown in Figure 5D. Treatment with CT20p proved toxic to these immortalized cells given their high expression levels of CCT2 (Figure 5A, 5D). Normal tissues, on the other hand, are not immortalized with oncogenes, so levels of CCT2 were lower, as we observed with normal lung tissue (Figures 1B, 5E) and tissues from other organs such as the heart and liver (Figure 5E). To examine any potential toxicities that could result from CT20p treatment, we performed a dose escalation study in mice. We observed no significant alternations in the liver and kidney function of mice and all clinical chemistry values were within reference ranges (Supplementary Figure 6), nor were any changes in weight, blood in urine or necrosis of organs detected (Supplementary Figure 6). Hence, our studies suggest that a cell's susceptibility to CT20p is based on the expression levels of CCT, as well as the activity of the chaperonin. With additional study of CCT in normal and cancer cells, such findings could lead to establishing a therapeutic window as the basis of an effective treatment strategy.

## DISCUSSION

In this study, tissues and cells from various cancers and normal organs were used to investigate CCT as a potential biomarker and therapeutic target in cancer treatment. We assessed CCT2 subunit protein levels across prostate, liver, and lung cancer tissue specimens and found that CCT2 expression levels correlated with disease progression in liver and prostate cancers, and CCT2 levels were significantly higher in lung carcinomas as compared to normal lung tissue independent of disease stage. We also showed that CCT2 levels were low in normal heart, liver, and lung tissues. These findings build upon our previous discoveries in breast and prostate cancer in which CCT2 levels were elevated as compared to non-disease tissues. Using SCLC cell lines, we demonstrated susceptibility to killing by our putative CCT inhibitor, CT20p, which proved more effective than a STAT3 inhibitor, and the resulting reduction of CCT client proteins (e.g., STAT3) in part addressed the mechanism of action for CT20p.

CCT belongs to the family of group II chaperonins that are found in eukaryotic cells and archaea and consists of eight paralogous subunits that differ mainly at the apical domains [24]. Unique to CCT is the formation of hetero-oligomeric complexes and the use of an ATP-dependent conformational change to control the opening/closing of a built-in lid [24, 53]. Hence, subunit diversity and sequential substrate binding generates multiple substrate binding sites that, when coupled to the lid closing mechanism, endow CCT with a flexibility to fold many more proteins than its bacterial counterparts [54]. This broad range of protein substrates means that 5-10% of newly synthesized proteins may require CCT for folding [44], but also that only part of the CCT interactome has been discovered. Given our findings of elevated CCT expression across cancer types, it is likely that CCT is responsible for folding more of the cell proteome in cancer or transformed cells than in normal cells. There also seems to be a selectivity for CCT subunits based on cancer types. For example, CCT3 and CCT8 were overexpressed in hepatocellular carcinoma (HCC) [28, 45, 55, 56] and the proteins were detected in the plasma from patients, suggesting that CCT3 could be used as a biomarker for screening HCC. We and others found that CCT2 was elevated in breast cancer [30, 31] as well as in gall bladder carcinoma [42]. In the current study, in addition to HCC and prostate cancer, we also found that CCT2 was increased in SCLC and in advanced stages of SqCLC. Understanding why cancers are selective for CCT subunits may require a better understanding of the basis of CCT substrate recognition as well as the discovery of new classes CCT substrates, such as those up-regulated in cancer cells.

As a possible CCT inhibitor, CT20p is one of the first. While arsenic was shown to inhibit CCT function and impair both actin and tubulin filaments [57], other than CT20p, only N-iodoacetyl-tryptophan (I-Trp), a synthetic small molecule, has been found to disrupt the interaction between CCT and tubulin, causing apoptosis through an ER stress-dependent mechanism [58, 59]. In contrast to I-Trp, CT20p does not cause death through a traditional apoptotic mechanism [33], which can be downregulated in cancer cells; rather, its inhibitory effects could extend to multiple client proteins that are essential for cancer progression [60]. Hence, inhibitors of protein folding have therapeutic value since their inhibition can decrease the pool of oncoproteins that cancer cells depend on. As an example, inhibitors of the heat shock protein family (HSPs), Hsp90 or Hsp70, showed significant efficacy in pre-clinical studies [61]. The Hsp90 inhibitor, Ganetespib, proved effective in SCLC cell lines, causing cell cycle arrest and caspase-3 dependent apoptosis [62]. However, human clinical trials in non-small cell lung cancer (NSCLC) with molecularly unselected patients were disappointing. While some promising results were observed in a cohort of patients with ALK-rearrangements, HSP inhibitors overall did not perform as expected from the pre-clinical data. The reasons for this could be that inhibition of HSPs may trigger a protective heat shock response through activation of heat shock transcription factor 1 (HSF1) and GRP78, which blocks the apoptosis of cancer cells. Alternatively, the level of HSP inhibition achieved in patients could have been insufficient to kill tumor cells [63]. Patients also developed resistance to Hsp90 inhibition due to activation of RAF/MEK/ERK or PI3K/AKT/MTOR pathways, requiring the need for combination approaches to make Hsp90 inhibitors work [64]. We anticipate that inhibition of CCT with CT20p may not result in similar problems because we have no evidence of induction of a heat shock response, such as the membrane translocation of GRP78 (data not shown). Further, the use of nanoparticles to deliver CT20p to tumors can achieve therapeutically relevant doses [32–34]. Our data also suggest that CT20p could knock out pathways—such as those induced through STAT3—that are linked to growth and survival signaling; hence, resistance activated by these pathways may not develop.

STAT3, which regulates the expression of genes involved in cancer progression, is an attractive target for inhibition in SCLC since it is found in primary SCLC tissues and in SCLC cell lines [65]. While a number of STAT3 inhibitors have been developed, most either inhibit the kinases that phosphorylate and activate STAT3 or they prevent the formation of STAT3 dimers through disruption of the protein's SH2 domains. Drugs, like the antibiotic doxycycline, inhibit STAT3 in lung cancer cells at the cost of severe side effects in patients like nausea, vomiting and diarrhea, rash and sensitivity to sun [66]. While there are many STAT3 inhibitors, few have moved into clinical trials and none to the clinic. Reasons for this failure are unknown but could be explained by the fact that most STAT3 inhibitors target the dimerization or phosphorylation domains; therefore, unphosphorylated STAT3 is unaffected and available for activation [67]. Since CCT is essential for the synthesis, refolding, and activity of STAT3 [37], inhibitors of CCT should also reduce the levels of total STAT3. Our data show that in SCLC cell lines, CT20p reduced STAT3 levels, while these same cell lines were resistant to killing with a STAT3 inhibitor. In fact, only one SCLC cell line was susceptible to the STAT3 inhibitor and also had the highest levels of STAT3. This cell line was also killed by CT20p, likely due to the loss of STAT3 along with other CCT client proteins.

Cancers like SCLC are challenging diseases to treat and are typically diagnosed at advanced stages. Systemic treatments are not curative [3]. Molecular targeted therapies are promising, despite requiring patients to be pre-screened, but only a few targets, like ALK, are associated with clinically relevant inhibitors (e.g., Crizotinib) and patients frequently relapse [63]. Our research suggests that therapeutically targeting CCT with inhibitors like CT20p could have benefits for SCLC patients. As shown in our studies, CCT subunits are overexpressed in SCLC as well as in advanced stages of other cancers. We anticipate that each patient will likely have their own unique CCT interactome based on the oncogenes that drive their cancer. The products of many of these oncogenes—such as STAT3, KRAS, or MYC—are CCT substrates; hence, each patient whose cancer overexpresses CCT could be treated without the need of molecular subtyping the downstream substrates. So whether a patient's tumor is driven by STAT3, MYC, Notch or a combination, they could still be treated with a CCT inhibitor since the chaperonin is essential for these proteins to reach functional status. Our approach, using the nanoparticle platform to deliver CT20p, has the potential to reach therapeutic doses in tumors and minimize off-site accumulation. CT20p treatment also has promising use in combination approaches with other drugs. Trials with checkpoint inhibitors in SCLC resulted in some durable responses [8], generating interest in agents like CT20p that could make cancers immunogenic or sensitive to radiation therapy.

## MATERIALS AND METHODS

### Immunohistochemistry

Tissue microarrays (TMAs) used in this study were obtained from US Biomax, Inc. Their catalog numbers are as follows: CO484a (colonic carcinoma), PR803b and PR631 (prostate carcinoma), BC03118 (hepatocellular carcinoma), LC726b, LC802a, LC802c, and BC041115a (lung carcinomas) and BN501 (normal tissue array). Each TMA contained varied numbers of patient tissue cores as well as normal tissue corresponding to the specific cancer type being analyzed. Please refer to Supplementary Tables 1, 2 and 5 for the number of samples per cancer type. Information about the tissue type, TNM, score, tumor grade, and stage were provided with the samples. TMAs were stained for CCT2 using anti-CCTβ antibody (LS-B4861; LifeSpan Biosciences). TMA LC802c was stained in parallel for CCT2 and Stat3 (anti-Stat3 antibody ab32500; Abcam). Primary antibodies were diluted 1:100 in Antibody Diluent (Leica). Staining of tissue arrays was performed by a Bond-Max Immunostainer (Leica), with an epitope retrieval buffer of EDTA pH 9.0 (Leica). Polymer Refine Detection reagents (Leica) were used, which include a hematoxylin counterstain. Image acquisition and scoring of staining was performed by a surgical pathologist as previously published [31].

### Cell lines and culture condition

NCIH1882 (ATCC CRL5903), NCIH1048 (ATCC CRL5853), NCIH1105 (ATCC CRL5856) and NCIH719 (ATCC CRL5837) were cultured in HITES medium supplemented with 5% FBS (Gemini). NCIH1417 (ATCC CRL5869) was cultured in RPMI-1640 in 10% FBS. MCF 10A (ATCC CRL10317) cells were cultured in Mammary Epithelial Cell Growth Media using MEGM bullet kit (Lonza). THLE2 (ATCC CRL2706) cells were cultured in Bronchial Epithelial Cell Growth Media using BEGM bullet kit (Lonza) without the supplied epinephrine, supplemented with an extra 5 ng/mL EGF (Corning), 70 ng/mL phosphoethanolamine (Sigma) and 10% FBS. AC16 Human Cardiomyocyte Cell Line (Millipore-Sigma) was cultured in AC16 expansion medium [49]. All media contained 1% antibiotic antimycotic solution (Corning). Cells were grown in a humidified 37° incubator with 5% carbon dioxide. All experiments with listed cell lines were performed within 4 months of receiving them using low passage number cells. Viability was routinely assessed by trypan blue exclusion (Gibco). Supplementary Table 2 provides additional cell line information as well as the lot number of all cells lines used in this study.

### Reagents

CT20p (VTIFVAGVLTASLTIWKKMG) was commercially synthesized (Biopeptide Co., Inc) at >98% purity, with the N- and C- terminals capped with acetyl and amine groups, respectively. For cellular delivery, CT20p was encapsulated in hyperbranched polyester nanoparticles (HBPE-NPs) as previously described [33, 34, 36]. Typically, a peptide loading of 0.15 μg CT20p to 1 mg polymer is achieved. Stat3 inhibitor VI S31-201 (Calbiochem) was purchased already in solution.

### Treatments

Cytotoxicity experiments: the indicated adherent cell lines were seeded in 96 well plates at the recommended seeding density and treated at 70-80% confluency. Suspension cell lines were transferred to a 96 well plates in 70% of final well volume, using fresh medium. Adherent cells were treated by replacing the well medium with medium containing CT20p-NPs or Stat3 inhibitor VI at final treatment concentration. Suspension cells were treated in 30μLs (3.34X concentration) 24 hours after being transferred to 96 well plate. The concentrations selected for CT20p-HBPE-NPs treatments were 75 μg nanoparticle/mL 150 μg nanoparticle/mL. The concentrations of Stat3 inhibitor VI (S31-201) used were 50μM and 100μM, based on product description overview regarding effective concentration of < 100μM (Calbiochem, 573130). NCIH1882 cells were treated for 16 hours with 150 μg/mL CT20p-NPs, in parallel with untreated control, which received medium only. Cells were lifted, washed in 1XPBS and frozen at -80°C until lysate preparation. NCIH1048 cells were treated for 6 hours before being lifted for lysate preparation.

### Measuring cell viability

Cytotoxicity of cells treated with CT20p-NPs or Stat3 inhibitor VI was determined by adding Calcein-AM and ethidium homodimer (Live/Dead Thermofisher) 48 hours post-treatment and measuring signal intensity using GFP and TexasRed filter cubes respectively. Signal intensity and image acquisition were obtained using Cytation5 (Biotek) and Gen5 software. Per well, three distinct, non-overlapping areas were imaged using a 4X phase objective without and with each appropriate filter. Because suspension cells grow in clusters, determining cell number using the “cellular analysis” feature is suboptimal. We used image analysis to determine GFP and TexasRed mean fluorescent intensity. Threshold values were employed when appropriate to eliminate background signal. All the data points generated per condition are averaged and used to calculate the ratio between intensity from GFP (live cells) and TexasRed (dead cells). Only unprocessed images were used for data analysis. Representative images are shown on figures.

### Immunoblotting

Cell pellets used to make protein lysates were washed in 1X PBS (Corning) and frozen at -80°C for at least 1 hour prior to lysis. Lysates were obtained by quickly adding ice cold NP-40 Lysis Buffer (50mM K^+^HEPES pH 7.5, 150mM NaCl, 1% NP-40, 1mM EDTA) with freshly added Halt protease inhibitor (Thermo, 78438) immediately after pellets were removed from the -80°C. Lysis took place on ice for 20 minutes with gentle and intermittent vortexing intervals. Lysates were cleared by centrifuging at 12,000 x g for 10 minutes, at 4°C. Soluble lysates were stored at -20°C for no longer than 3 months. Freeze/thaw cycles were minimized to avoid protein precipitation and degradation. Proteins in the lysates were resolved by SDS-PAGE and transferred to Immobilon-FL PVDF membrane (Millipore IPFL00010). Prior to blocking, membranes were stained for total protein using Revert (LI-COR) and imaged on Odyssey (LI-COR) using the 700 channel. After blocking, blots were probed with primary antibodies against CCT2 (anti-CCTβ, Millipore), CCT4 (anti-CCTΔ, Abcam), CCT5 (anti-CCTε, Abcam), and STAT3 (anti-Stat3, Abcam), followed by 1-hour incubation with secondary antibodies IRDye 800CW and/or IRDye 680CW (LI-COR) at a 1:10000 dilution. Bands were visualized by NIR fluorescence detection using Odyssey (LI-COR). Relative protein quantification was performed with Image Studio software (LI-COR). The signal obtained for proteins of interest were normalized to total protein content using Revert (LI-COR) image as follows: first, total protein quantification was performed using the 700nm channel, using all the protein transferred onto the blot after Revert staining. Second, lane normalization factor (LNF) was calculated by dividing the signal of each lane by the signal from the lane with highest signal. Third, target signal was determined. Normalization was performed by dividing the signal from each target by the LNS. Immunostaining was performed with a minimum of two biological replicates and at least three technical replicates per target.

### Statistical analysis

Experiments were performed at least three times, in duplicates or triplicates, where appropriate. Data representative of experiments were selected for this publication. TMA data is expressed as means and standard deviation, whereas blots and Live/Dead assay data is represented as means and standard error. One-way ANOVA was used to compare mean scoring between the different groups defined by various tissue parameters as well as signal intensity ratio in treated cells. Tukey's multiple comparison test was used to compare significance between individual groups. For CT20p treatment analyzed by immunoblot, unpaired, two-tailed t-test was used to calculate significance between untreated and treated samples. Calculations were performed with GraphPad Prism software (GraphPad). Statistical significance was defined as p < 0.05.

Datasets generated in the current study are available in the Kaplan-Meier Plotter (KMPlot) repository [68, 69] at http://kmplot.com/analysis/index.php?p=service&cancer=breast.

## SUPPLEMENTARY MATERIALS FIGURES AND TABLES



## Abbreviations

CCT or TRiC: Chaperonin-Containing TCP-1 or T-complex 1 ring protein
CT20p-NPs: CT20p peptide encapsulated in nanoparticles
EMT: Epithelial-to-mesenchymal
HSPs: Heat shock protein family
HCC: Heptacellular carcinoma
NPs: Nanoparticles
I-Trp: N-iodoacetyl-tryptophan
SCLC: Small cell lung cancer
SqCLC: Squamous cell lung carcinoma
TMAs: Tissue microarrays
WT: Wild-type

## References

[1] Howlader N, Noone AM, Krapcho M, Miller D, Bishop K, Kosary CL, Yu M, Ruhl J, Tatalovich Z, Mariotto A, Lewis DR, Chen HS, Feuer EJ, Cronin KA (2016). SEER Cancer Statistics Review, 1975-2014.

[2] Carter BW, Glisson BS, Truong MT, Erasmus JJ (2014). Small cell lung carcinoma: staging, imaging, and treatment considerations. Radiographics.

[3] Waqar SN, Morgensztern D (2017). Treatment advances in small cell lung cancer (SCLC). Pharmacol Ther.

[4] Arnedos M, Bihan C, Delaloge S, Andre F (2012). Triple-negative breast cancer: are we making headway at least?. Ther Adv Med Oncol.

[5] Sørensen M, Pijls-Johannesma M, Felip E, ESMO Guidelines Working Group (2010). Small-cell lung cancer: ESMO Clinical Practice Guidelines for diagnosis, treatment and follow-up. Ann Oncol.

[6] Demedts IK, Vermaelen KY, van Meerbeeck JP (2010). Treatment of extensive-stage small cell lung carcinoma: current status and future prospects. Eur Respir J.

[7] Bramati A, Girelli S, Torri V, Farina G, Galfrascoli E, Piva S, Moretti A, Dazzani MC, Sburlati P, La Verde NM (2014). Efficacy of biological agents in metastatic triple-negative breast cancer. Cancer Treat Rev.

[8] Tartarone A, Giordano P, Lerose R, Rodriquenz MG, Conca R, Aieta M (2017). Progress and challenges in the treatment of small cell lung cancer. Med Oncol.

[9] Roviello G, Sobhani N, Generali D (2017). Bevacizumab in small cell lung cancer. Ann Transl Med.

[10] Ma X, Wang X, Huang J, Chen Y, Zhang J, Zhang B, Shi C, Liu L (2016). Bevacizumab addition in neoadjuvant treatment increases the pathological complete response rates in patients with HER-2 negative breast cancer especially triple negative breast cancer: a meta-analysis. PLoS One.

[11] Stratigos M, Matikas A, Voutsina A, Mavroudis D, Georgoulias V (2016). Targeting angiogenesis in small cell lung cancer. Transl Lung Cancer Res.

[12] Peifer M, Fernández-Cuesta L, Sos ML, George J, Seidel D, Kasper LH, Plenker D, Leenders F, Sun R, Zander T (2012). Integrative genome analyses identify key somatic driver mutations of small-cell lung cancer. Nat Genet.

[13] Santarpia M, Daffinà MG, Karachaliou N, González-Cao M, Lazzari C, Altavilla G, Rosell R (2016). Targeted drugs in small-cell lung cancer. Transl Lung Cancer Res.

[14] Tatematsu A, Shimizu J, Murakami Y, Horio Y, Nakamura S, Hida T, Mitsudomi T, Yatabe Y (2008). Epidermal growth factor receptor mutations in small cell lung cancer. Clin Cancer Res.

[15] Kijima T, Maulik G, Ma PC, Tibaldi EV, Turner RE, Rollins B, Sattler M, Johnson BE, Salgia R (2002). Regulation of cellular proliferation, cytoskeletal function, signal transduction through CXCR4 and c-Kit in small cell lung cancer cells. Cancer Res.

[16] Maulik G, Madhiwala P, Brooks S, Ma PC, Kijima T, Tibaldi EV, Schaefer E, Parmar K, Salgia R (2002). Activated c-Met signals through PI3K with dramatic effects on cytoskeletal functions in small cell lung cancer. J Cell Mol Med.

[17] Maulik G, Kijima T, Ma PC, Ghosh SK, Lin J, Shapiro GI, Schaefer E, Tibaldi E, Johnson BE, Salgia R (2002). Modulation of the c-Met/hepatocyte growth factor pathway in small cell lung cancer. Clin Cancer Res.

[18] Lee JH, Kang KW, Kim JE, Hwang SW, Park JH, Kim SH, Ji JH, Kim TG, Nam HY, Roh MS, Lee EH, Park MI, Kim MS, Lee HW (2015). Differential expression of heat shock protein 90 isoforms in small cell lung cancer. Int J Clin Exp Pathol.

[19] Byers LA, Wang J, Nilsson MB, Fujimoto J, Saintigny P, Yordy J, Giri U, Peyton M, Fan YH, Diao L, Masrorpour F, Shen L, Liu W (2012). Proteomic profiling identifies dysregulated pathways in small cell lung cancer and novel therapeutic targets including PARP1. Cancer Discov.

[20] Xu L, Wu X, Hu C, Zhang Z, Zhang L, Liang S, Xu Y, Zhang F (2016). A meta-analysis of combination therapy versus single-agent therapy in anthracycline- and taxane-pretreated metastatic breast cancer: results from nine randomized Phase III trials. Onco Targets Ther.

[21] Komarova NL, Boland CR (2013). Cancer: calculated treatment. Nature.

[22] Leitner A, Joachimiak LA, Bracher A, Mönkemeyer L, Walzthoeni T, Chen B, Pechmann S, Holmes S, Cong Y, Ma B, Ludtke S, Chiu W, Hartl FU (2012). The molecular architecture of the eukaryotic chaperonin TRiC/CCT. Structure.

[23] Narayanan A, Pullepu D, Kabir MA (2016). The interactome of CCT complex - a computational analysis. Comput Biol Chem.

[24] Spiess C, Meyer AS, Reissmann S, Frydman J (2004). Mechanism of the eukaryotic chaperonin: protein folding in the chamber of secrets. Trends Cell Biol.

[25] Cong Y, Schröder GF Meyer AS, Jakana J, Ma B, Dougherty MT, Schmid MF, Reissmann S, Levitt M, Ludtke SL, Frydman J, Chiu W (2012). Symmetry-free cryo-EM structures of the chaperonin TRiC along its ATPase-driven conformational cycle. EMBO J.

[26] Meyer AS, Gillespie JR, Walther D, Millet IS, Doniach S, Frydman J (2003). Closing the folding chamber of the eukaryotic chaperonin requires the transition state of ATP hydrolysis. Cell.

[27] Boudiaf-Benmammar C, Cresteil T, Melki R (2013). The cytosolic chaperonin CCT/TRiC and cancer cell proliferation. PLoS One.

[28] Huang X, Wang X, Cheng C, Cai J, He S, Wang H, Liu F, Zhu C, Ding Z, Huang X, Zhang T, Zhang Y (2014). Chaperonin containing TCP1, subunit 8 (CCT8) is upregulated in hepatocellular carcinoma and promotes HCC proliferation. APMIS.

[29] Yokota S, Yamamoto Y, Shimizu K, Momoi H, Kamikawa T, Yamaoka Y, Yanagi H, Yura T, Kubota H (2001). Increased expression of cytosolic chaperonin CCT in human hepatocellular and colonic carcinoma. Cell Stress Chaperones.

[30] Guest ST, Kratche ZR, Bollig-Fischer A, Haddad R, Ethier SP (2015). Two members of the TRiC chaperonin complex, CCT2 and TCP1 are essential for survival of breast cancer cells and are linked to driving oncogenes. Exp Cell Res.

[31] Bassiouni R, Nemec KN, Iketani A, Flores O, Showalter A, Khaled AS, Vishnubhotla P, Sprung RW, Kaittanis C, Perez JM, Khaled AR (2016). Chaperonin containing TCP-1 protein level in breast cancer cells predicts therapeutic application of a cytotoxic peptide. Clin Cancer Res.

[32] Flores O, Santra S, Kaittanis C, Bassiouni R, Khaled AS, Khaled AR, Grimm J, Perez JM (2017). PSMA-targeted theranostic nanocarrier for prostate cancer. Theranostics.

[33] Boohaker RJ, Zhang G, Lee MW, Nemec KN, Santra S, Perez JM, Khaled AR (2012). Rational development of a cytotoxic peptide to trigger cell death. Mol Pharm.

[34] Lee MW, Bassiouni R, Sparrow NA, Iketani A, Boohaker RJ, Moskowitz C, Vishnubhotla P, Khaled AS, Oyer J, Copik A, Fernandez-Valle C, Perez JM, Khaled AR (2014). The CT20 peptide causes detachment and death of metastatic breast cancer cells by promoting mitochondrial aggregation and cytoskeletal disruption. Cell Death Dis.

[35] Santra S, Kaittanis C, Perez JM (2010). Cytochrome C encapsulating theranostic nanoparticles: a novel bifunctional system for targeted delivery of therapeutic membrane-impermeable proteins to tumors and imaging of cancer therapy. Mol Pharm.

[36] Santra S, Kaittanis C, Perez JM (2010). Aliphatic hyperbranched polyester: a new building block in the construction of multifunctional nanoparticles and nanocomposites. Langmuir.

[37] Kasembeli M, Lau WC, Roh SH, Eckols TK, Frydman J, Chiu W, Tweardy DJ (2014). Modulation of STAT3 folding and function by TRiC/CCT chaperonin. PLoS Biol.

[38] Kamran MZ, Patil P, Gude RP (2013). Role of STAT3 in cancer metastasis and translational advances. Biomed Res Int.

[39] Zhao X, Sun X, Li XL (2012). Expression and clinical significance of STAT3, P-STAT3, VEGF-C in small cell lung cancer. Asian Pac J Cancer Prev.

[40] Lauwers GY, Terris B, Balis UJ, Batts KP, Regimbeau JM, Chang Y, Graeme-Cook F, Yamabe H, Ikai I, Cleary KR, Fujita S, Flejou JF, Zukerberg LR (2002). Prognostic histologic indicators of curatively resected hepatocellular carcinomas: a multi-institutional analysis of 425 patients with definition of a histologic prognostic index. Am J Sur Pathol.

[41] American Cancer Society (2017). Cancer Facts & Figures.

[42] Zou Q, Yang ZL, Yuan Y, Li JH, Liang LF, Zeng GX, Chen SL (2013). Clinicopathological features and CCT2 and PDIA2 expression in gallbladder squamous/adenosquamous carcinoma and gallbladder adenocarcinoma. World J Surg Oncol.

[43] D’Amico D, Carbone D, Mitsudomi T, Nau M, Fedorko J, Russell E, Johnson B, Buchhagen D, Bodner S, Phelps R (1992). High frequency of somatically acquired p53 mutations in small-cell lung cancer cell lines and tumors. Oncogene.

[44] Yam AY, Xia Y, Lin HT, Burlingame A, Gerstein M, Frydman J (2008). Defining the TRiC/CCT interactome links chaperonin function to stabilization of newly made proteins with complex topologies. Nat Struct Mol Biol.

[45] Zhang Y, Wang Y, Wei Y, Wu J, Zhang P, Shen S, Saiyin H, Wumaier R, Yang X, Wang C, Yu L (2016). Molecular chaperone CCT3 supports proper mitotic progression and cell proliferation in hepatocellular carcinoma cells. Cancer Lett.

[46] Lin L, Amin R, Gallicano GI, Glasgow E, Jogunoori W, Jessup JM, Zasloff M, Marshall JL, Shetty K, Johnson L, Mishra L, He AR (2009). The STAT3 inhibitor NSC 74859 is effective in hepatocellular cancers with disrupted TGF-beta signaling. Oncogene.

[47] Siddiquee K, Zhang S, Guida WC, Blaskovich MA, Greedy B, Lawrence HR, Yip ML, Jove R, McLaughlin MM, Lawrence NJ, Sebti SM, Turkson J (2007). Selective chemical probe inhibitor of Stat3, identified through structure-based virtual screening, induces antitumor activity. Proc Natl Acad Sci U S A.

[48] De Angelis A, Urbanek K, Cappetta D, Piegari E, Pia Ciuffreda L, Rivellino A, Russo R, Esposito G, Rossi F, Berrino L (2016). Doxorubicin cardiotoxicity and target cells: a broader perspective. Cardiooncology.

[49] Davidson MM, Nesti C, Palenzuela L, Walker WF, Hernandez E, Protas L, Hirano M, Isaac ND (2005). Novel cell lines derived from adult human ventricular cardiomyocytes. J Mol Cell Cardiol.

[50] Pfeifer AM, Cole KE, Smoot DT, Weston A, Groopman JD, Shields PG, Vignaud JM, Juillerat M, Lipsky MM, Trump BF (1993). Simian virus 40 large tumor antigen-immortalized normal human liver epithelial cells express hepatocyte characteristics and metabolize chemical carcinogens. Proc Natl Acad Sci U S A.

[51] Meuwissen R, Linn SC, Linnoila RI, Zevenhoven J, Mooi WJ, Berns A (2003). Induction of small cell lung cancer by somatic inactivation of both Trp53 and Rb1 in a conditional mouse model. Cancer Cell.

[52] Furukawa M, Wheeler S, Clark AM, Wells A (2015). Lung epithelial cells induce both phenotype alteration and senescence in breast cancer cells. PLoS One.

[53] Spiess C, Miller EJ, McClellan AJ, Frydman J (2006). Identification of the TRiC/CCT substrate binding sites uncovers the function of subunit diversity in eukaryotic chaperonins. Mol Cell.

[54] Joachimiak LA, Walzthoeni T, Liu CW, Aebersold R, Frydman J (2014). The structural basis of substrate recognition by the eukaryotic chaperonin TRiC/CCT. Cell.

[55] Cui X, Hu ZP, Li Z, Gao PJ, Zhu JY (2015). Overexpression of chaperonin containing TCP1, subunit 3 predicts poor prognosis in hepatocellular carcinoma. World J Gastroenterol.

[56] Qian EN, Han SY, Ding SZ, Lv X (2016). Expression and diagnostic value of CCT3 and IQGAP3 in hepatocellular carcinoma. Cancer Cell Int.

[57] Pan X, Reissman S, Douglas NR, Huang Z, Yuan DS, Wang X, McCaffery JM, Frydman J, Boeke JD (2010). Trivalent arsenic inhibits the functions of chaperonin complex. Genetics.

[58] Lin YF, Tsai WP, Liu HG, Liang PH (2009). Intracellular beta-tubulin/chaperonin containing TCP1-beta complex serves as a novel chemotherapeutic target against drug-resistant tumors. Cancer Res.

[59] Lin YF, Lee YF, Liang PH (2012). Targeting beta-tubulin: CCT-beta complexes incurs Hsp90- and VCP-related protein degradation and induces ER stress-associated apoptosis by triggering capacitative Ca2+ entry, mitochondrial perturbation and caspase overactivation. Cell Death Dis.

[60] Lee MW, Bassiouni R, Iketani A, Flores O, Perez JM, Khaled AR (2014). The CT20 peptide: more than a piece of bax. Cancer Cell Microenviron.

[61] Zheng Y, Yamaguchi H, Tian C, Lee MW, Tang H, Wang HG, Chen Q (2005). Arsenic trioxide (As(2)O(3)) induces apoptosis through activation of Bax in hematopoietic cells. Oncogene.

[62] Lai CH, Park KS, Lee DH, Alberobello AT, Raffeld M, Pierobon M, Pin E, Petricoin Iii EF, Wang Y, Giaccone G (2014). HSP-90 inhibitor ganetespib is synergistic with doxorubicin in small cell lung cancer. Oncogene.

[63] Gainor JF, Dardaei L, Yoda S, Friboulet L, Leshchiner I, Katayama R, Dagogo-Jack I, Gadgeel S, Schultz K, Singh M, Chin E, Parks M, Lee D (2016). Molecular mechanisms of resistance to first- and second-generation ALK inhibitors in ALK-rearranged lung cancer. Cancer Discov.

[64] Chatterjee S, Huang EH, Christie I, Kurland BF, Burns TF (2017). Acquired resistance to the Hsp90 inhibitor, ganetespib, in KRAS-mutant NSCLC is mediated via reactivation of the ERK-p90RSK-mTOR signaling network. Mol Cancer Ther.

[65] Pfeiffer M, Hartmann TN, Leick M, Catusse J, Schmitt-Graeff A, Burger M (2009). Alternative implication of CXCR4 in JAK2/STAT3 activation in small cell lung cancer. Br J Cancer.

[66] Qin Y, Zhang Q, Lee S, Zhong WL, Liu YR, Liu HJ, Zhao D, Chen S, Xiao T, Meng J, Jing XS, Wang J, Sun B (2015). Doxycycline reverses epithelial-to-mesenchymal transition and suppresses the proliferation and metastasis of lung cancer cells. Oncotarget.

[67] Huang W, Dong Z, Chen Y, Wang F, Wang CJ, Peng H, He Y, Hangoc G, Pollok K, Sandusky G, Fu XY, Broxmeyer HE, Zhang ZY (2016). Small-molecule inhibitors targeting the DNA-binding domain of STAT3 suppress tumor growth, metastasis and STAT3 target gene expression in vivo. Oncogene.

[68] Szasz AM, Lánczky A, Nagy Á, Förster S, Hark K, Green JE, Boussioutas A, Busuttil R, Szabó A, Győrffy B (2016). Cross-validation of survival associated biomarkers in gastric cancer using transcriptomic data of 1,065 patients. Oncotarget.

[69] Gyorffy B, Lanczky A, Eklund AC, Denkert C, Budczies J, Li Q, Szallasi Z (2010). An online survival analysis tool to rapidly assess the effect of 22,277 genes on breast cancer prognosis using microarray data of 1,809 patients. Breast Cancer Res Treat.

