# Supplementary material for: Targeting chaperonin containing TCP1 (CCT) as a molecular therapeutic for small cell lung cancer
Source: Oncotarget. 2017 Nov 25;8(66):110273–88. doi: 10.18632/oncotarget.22681 (PMC5746381; doi:10.18632/oncotarget.22681)
Supplement: Supplementary file 1 [file oncotarget-08-110273-s001.pdf]

Targeting chaperonin containing TCP1 (CCT) as a molecular therapeutic for small cell lung cancer

SUPPLEMENTARY MATERIALS

Cell lines and culture conditions

BEAS2B (ATCC- CRL9609) was cultured using BEGM bullet kit (Lonza) according to ATCC specifications. Primary Human Normal Bronchial Epithelial (NHBE) cells (CC-2540 lot#0000580582) and Human Normal Lung Fibroblasts (NHLF) cells (CC-2512 lot#0000543644) were purchased from Lonza and cultured using BEGM and FGM-2 bullet kits respectively, according to manufacturer's specifications. Passage number 1, 2 or 3 were used for the experiments listed.

Immunohistochemistry

Tissue microarrays (TMAs) were purchased from US Biomax, Inc. Their catalog numbers are as follows: CO484a (colonic carcinoma), PR803b and PR631 (prostate carcinoma), BC03118 (hepatocellular carcinoma). Each TMA contained varied numbers of patient tissue cores as well as normal tissue corresponding to the specific cancer type being analyzed. Please refer to Supplementary Table 2 for number of samples per cancer type. Information about the tissue type, TNM, score, tumor grade, and stage were provided with the samples. TMAs were stained for CCT2 using anti-CCTβ antibody (LS-B4861; LifeSpan Biosciences) diluted 1:100 in Antibody Diluent (Leica). Staining of tissue arrays was performed by a Bond-Max Immunostainer (Leica), with an epitope retrieval buffer of EDTA pH 9.0 (Leica). Polymer Refine Detection reagents (Leica) were used, which include a hematoxylin counterstain. Scoring of staining was performed by a surgical pathologist as previously published [1].

Immunoblotting

Immunoblotting for p53 was performed as in Materials and Methods using anti-p53 antibody (Abcam). Protein levels were normalized to GAPDH (Santa Cruz).

Statistical analysis and data mining

Data representative of experiments were selected for this publication. TMA data is expressed as means and standard deviation. One-way ANOVA was used to compare mean scoring between the different groups defined by various tissue parameters as well as signal intensity ratio in treated cells. Tukey's multiple comparison test was used to compare significance between individual groups. Calculations were performed with GraphPad Prism software (GraphPad). Statistical significance was defined as $p < 0.05$. Survival data for lung cancer patients' expression high and low levels of CCT2 and CCT4 were queried using the TCGA database through cBioPortal.

For hepatocellular carcinoma: cBioPortal: Liver Hepatocellular carcinoma (AMC. Hepatology, 2014) 231 samples /1 gene. *For prostate carcinoma*: cBioPortal: Prostate Adenocarcinoma, Metastatic (Michigan, Nature 2012) 61 samples/ 1gene. For all Human Protein Atlas (HCC, prostate and colon carcinoma) data please refer to [2].

Toxicity

Three male and three female nude mice (6-8 weeks old) were treated with five doses ((2.4 mg/kg, 4.8 mg/kg, 9.6 mg/kg, 19.2 mg/kg, 38.4 mg/kg) of CT20p-NPs over a two week period. Treatments were performed on Days 0, 3, 7, 10 and 14 and mice were euthanized 14 days after the last treatment. Urine analysis was performed during treatment as described in Supplementary Figure 5. Results from a representative mouse are shown. Mice were terminally bleed and serum recovered two weeks after the last injection. Serum was analyzed for markers of liver and kidney function. Representative parameters are shown in comparison to a normal, untreated mouse. The weight of mice during treatment was determined. At the study endpoint, mice were sacrificed and blood collected to isolate serum. Serum was sent to Idexx for testing for the chemical chemistry panel which assesses kidney and liver function. No statistical differences were observed between control and NP-treated mice. All animal work followed the study protocol approved by the Institutional Animal Care and Use Committee (IACUC) at the University of Central Florida.

REFERENCES

1. Bassiouni R, Nemec KN, Iketani A, Flores O, Showalter A, Khaled AS, Vishnubhotla P, Sprung RW Jr, Kaittanis C, Perez JM, Khaled AR. Chaperonin containing TCP-1 protein level in breast cancer cells predicts therapeutic application of a cytotoxic peptide. Clin Cancer Res. 2016; 22: 4366-79.
2. Uhlen M, Zhang C, Lee S, Sjöstedt E, Fagerberg L, Bidkhori G, Benfeitas R, Arif M, Liu Z, Edfors F, Sanli K, von Feilitzen K, Oksvold P, et al. A pathology atlas of the human cancer transcriptome. Science. 2017.

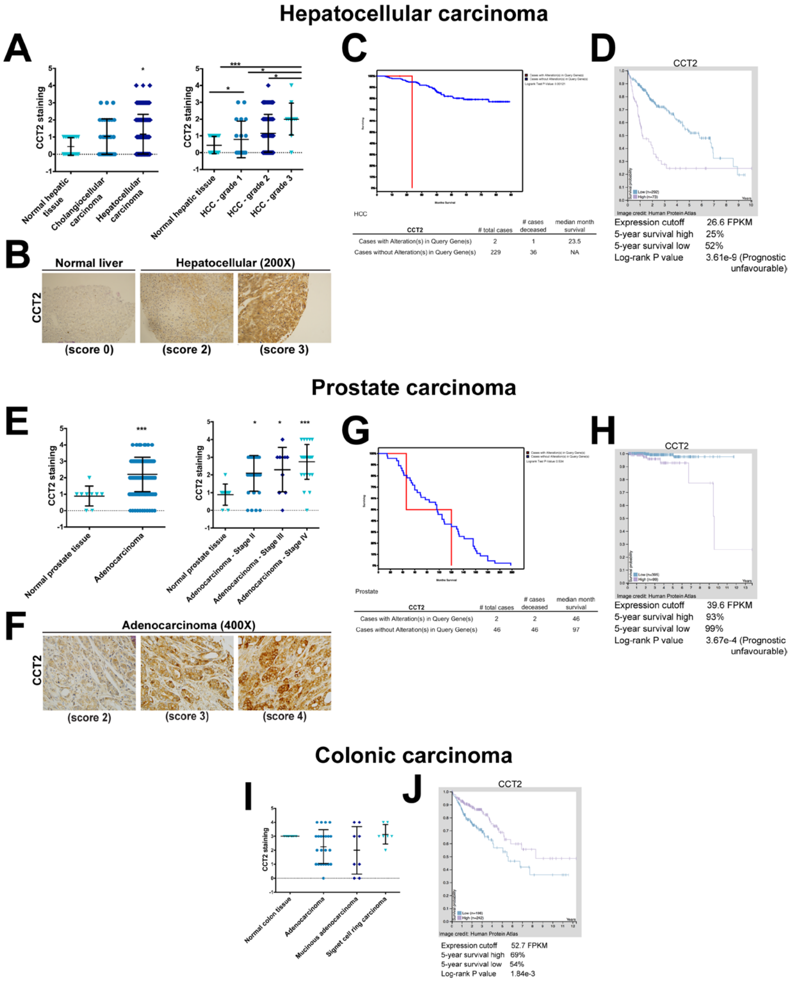

Supplementary Figure 1: Effects of high levels of CCT2 in hepatocellular, prostate and colon cancers. (A, E and **I)** Levels of CCT2 were assayed in human tumor tissue samples of (A) hepatocellular, (E) prostate and (I) colon cancer by immunohistochemistry (IHC) as described in Supplementary Methods and Materials. Representative images of different staining intensities are shown for **(D)** hepatocellular (200X) and **(H)** prostate (400X) tissues. Images of stained tissues as well as analysis of staining intensity were performed by a pathologist. For staining analysis, a score between 0-4 was give following a scheme previously published in Bassiouni et al (2016). Significance was calculated in reference to normal tissue. p<0.05, ** = p<0.01, *** = p<0.001, **** = p<0.0001. **(B, F)** Survival data for patients with high and low levels of CCT2 were queried using the TCGA database through cBioPortal (repository can be found in Supplementary Methods and Materials). (B) Kaplan-Meyer plot for HCC patients showing that duplication of CCT2 gene decreases survival. (F) Kaplan-Meyer plot for prostate cancer patients showing that genomic alterations in CCT2 decreases median survival rate by half. **(C, G** and **J)** Survival data for patients with high levels of CCT2 was queried using The Human Protein Atlas database showing that (C) in HCC patients with high levels of CCT2 the 5-year survival rate is 25% compared to 52% in patients with low levels of CCT2. (G) In prostate cancer, patients with high levels of CCT2 have a 5-year survival rate of 93% and patients with low levels of CCT2 have 99% survival rate. (J) In colonic carcinoma, the 5-year survival rate for patients with high levels of CCT2 is 69% versus 54% for patients with low levels.

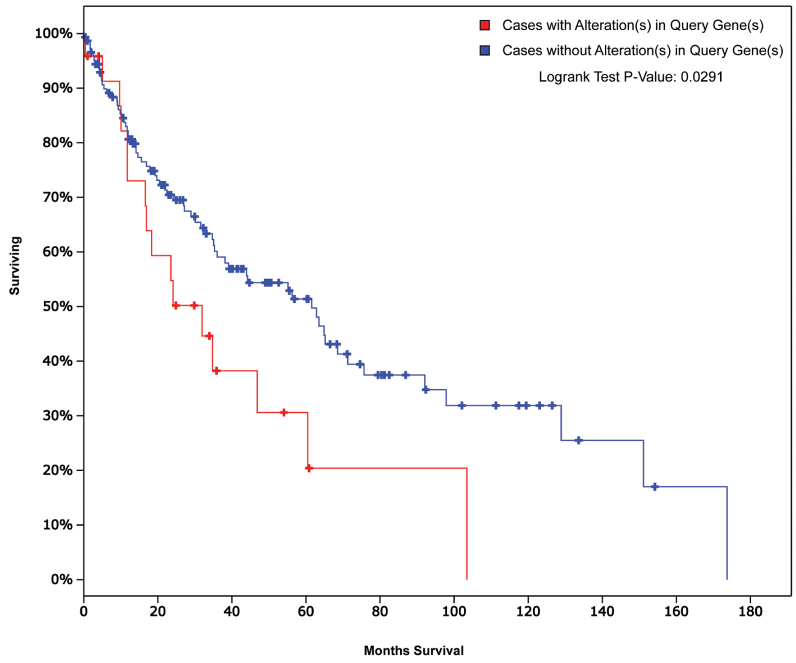

CCT2 and CCT4	# total cases	# cases deceased	median month survival
Cases with Alteration(s) in Query Gene(s)	24	16	31.96
Cases without Alteration(s) in Query Gene(s)	150	68	61.56

CCT2	# total cases	# cases deceased	median month survival
Cases with Alteration(s) in Query Gene(s)	5	4	18.36
Cases without Alteration(s) in Query Gene(s)	169	80	55.16

CCT4	# total cases	# cases deceased	median month survival
Cases with Alteration(s) in Query Gene(s)	19	12	31.96
Cases without Alteration(s) in Query Gene(s)	155	72	61.56

Supplementary Figure 2: Lung cancer patients with high levels of CCT2 and CCT4 have lower survival rates. Survival of patients with squamous cell lung carcinoma expressing high and low levels of CCT2 and CCT4 were queried using TCGA database through cBIoPortal. **(A)** Kaplan-Meyer plot of lung cancer patients shows that high levels of CCT2 and CCT4 are associated with shorter life expectancies. **(B)** Table showing summary of results for lung cancer patients with high and low levels of CCT2 and CCT4 individually. Although no statically significant due to the low number of patients, having high levels of CCT2 alone decreases survival rate by a factor of 3, while having high levels of CCT4 alone decreases survival rate by a factor of 2.

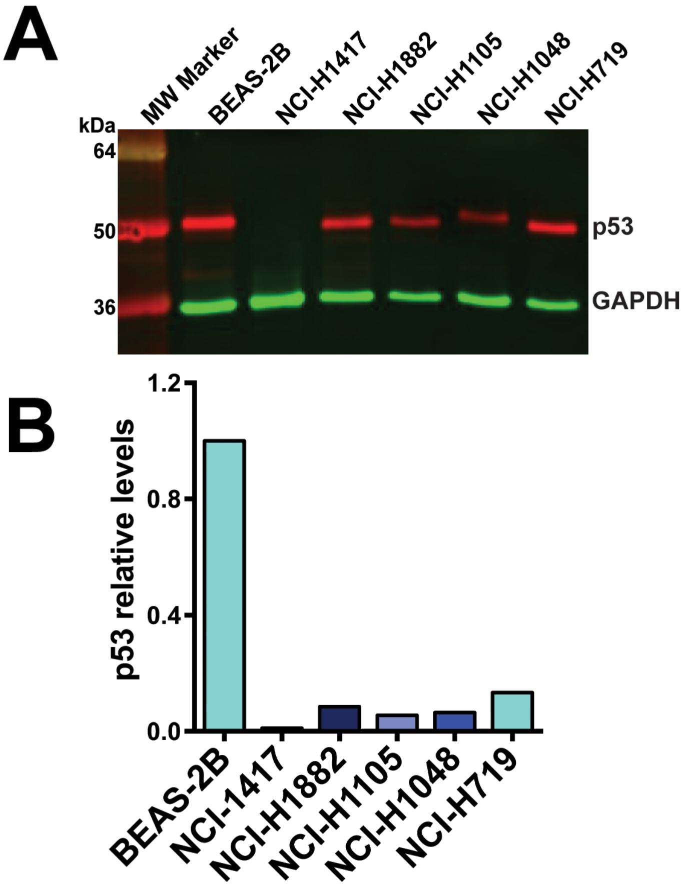

C

Cell line	p53 AA mutation
NCI-H1417 (ATCC CRL-5869)	R175L
NCI-H1882 (ATCC CRL-5903)	WT
NCI-H1105 (ATCC CRL-5856)	R249S
NCI-H1048 (ATCC CRL-5853)	R273C
NCI-H719 (ATCC CRL-5837)	R248Q

Supplementary Figure 3: Levels of p53 in SCLC cell lines. Cell lines from the ATCC SCLC p53 Hotspot mutation panel, used in this study, were assayed for p53 levels as quality control. **(A)** Total soluble lysate corresponding to indicated cell lines (five human SCLC cell lines and an immortalized lung breast epithelial cell line) were assayed for p53 levels (red band). GAPDH was used as loading control (green band). **(B)** Bar graph showing relative quantification of p53 levels which were normalized to GAPDH levels as described in Supplementary Methods and Materials. **(C)** Amino acid change due to genomic mutations found in TP53 for each cell line. All mutations resulted in "loss of function".

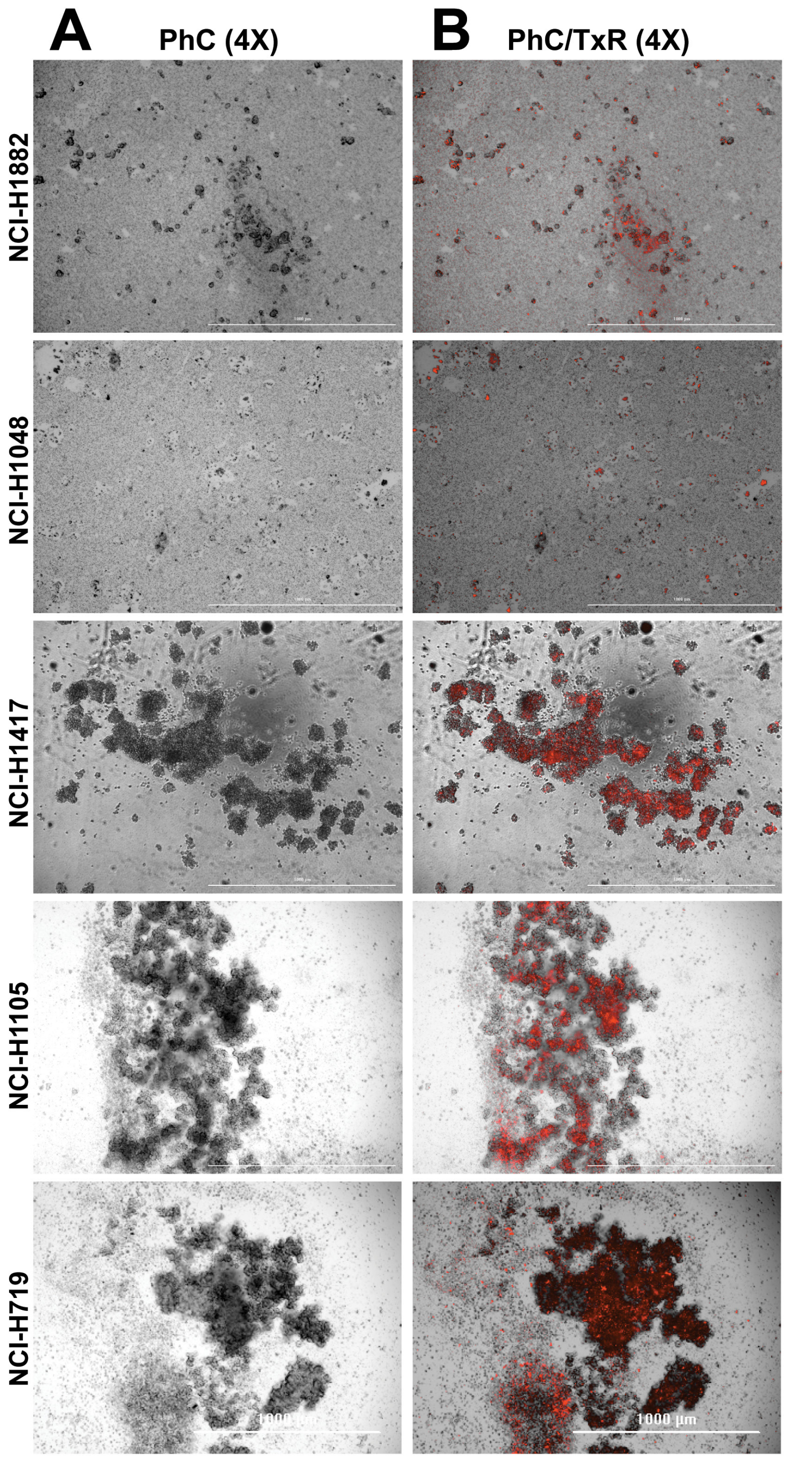

PhC - Phase Contrast
TxR - Texas Red filter

Supplementary Figure 4: CT20p-NPs uptake by SCLC cell lines. DiI dye (1,1'-Dioctadecyl-3,3,3',3'-Tetramethylindocarbocyanine Perchlorate) encapsulated in HBPE-nanoparticles were delivered to viable SCLC cells as described in Materials and Methods. Cells were imaged after 24 hours for fluorescent signal indicative of nanoparticle uptake. Column **(A)** Phase contrast images(4X) **(B)** Phase contrast(4X)/ TexasRed merged images.

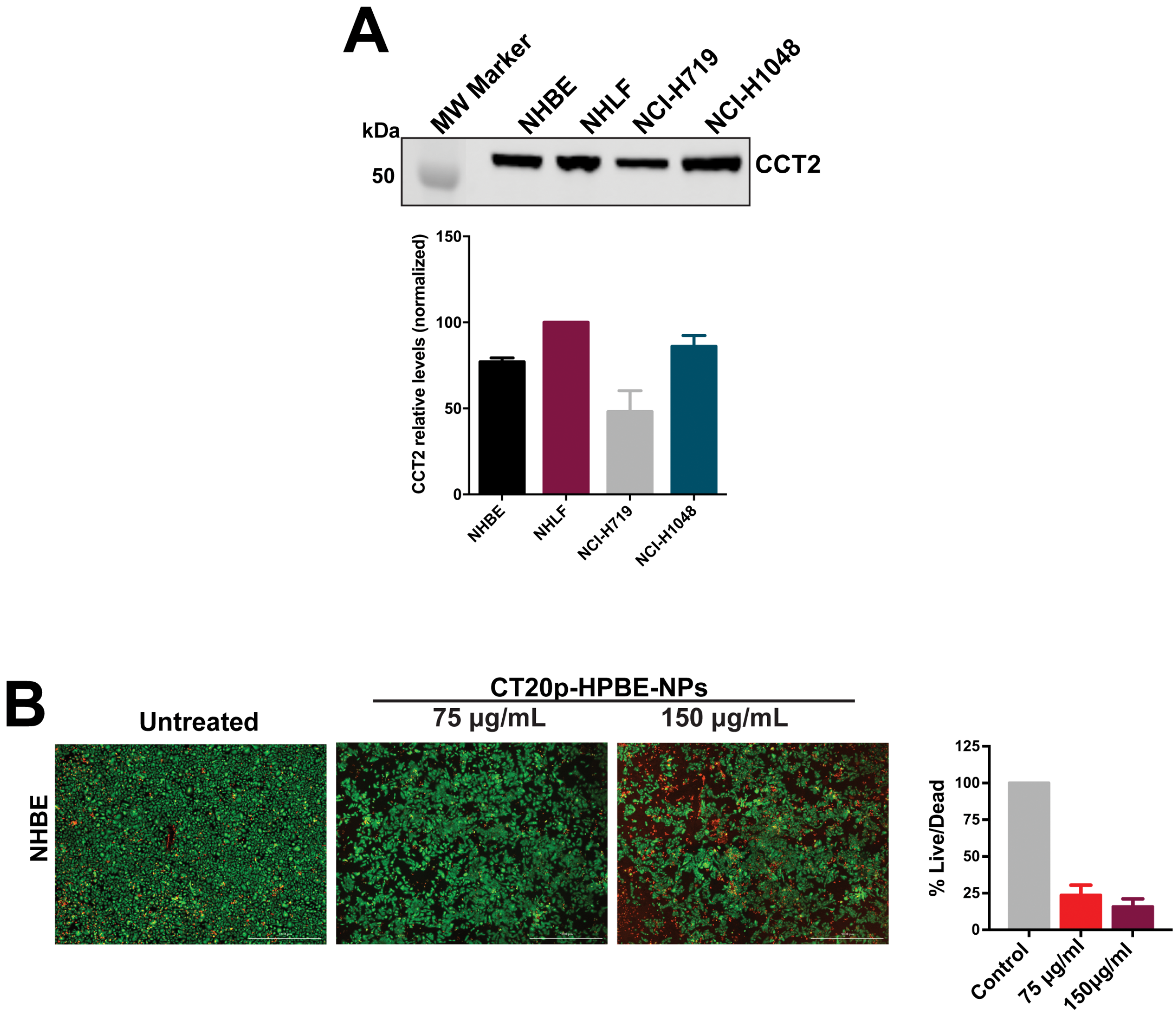

Supplementary Figure 5: Primary lung cells have variable levels of CCT2. Human normal bronchial epithelial cells (HNBE) as well as human normal lung fibroblasts were assayed for levels of CCT2 and susceptibility to CT20p. **(A)** Levels of CCT2 in primary lung cells were high compared to SCLC cell lines. Levels are shown as percent of the highest signal (HNLF) and normalization was performed as described in Figure 1. **(B)** NHBE cells were treated with CT20p as described in Methods and Materials and cytotoxicity detected using Live/Dead (Promega). Signal from 3 wells were averaged as means and standard error. Per well, four overlapping quadrants were imaged and stitched using Gen5 software. Images shown are representative of the experiment. Bar graph was generated as in Figure 3.

A

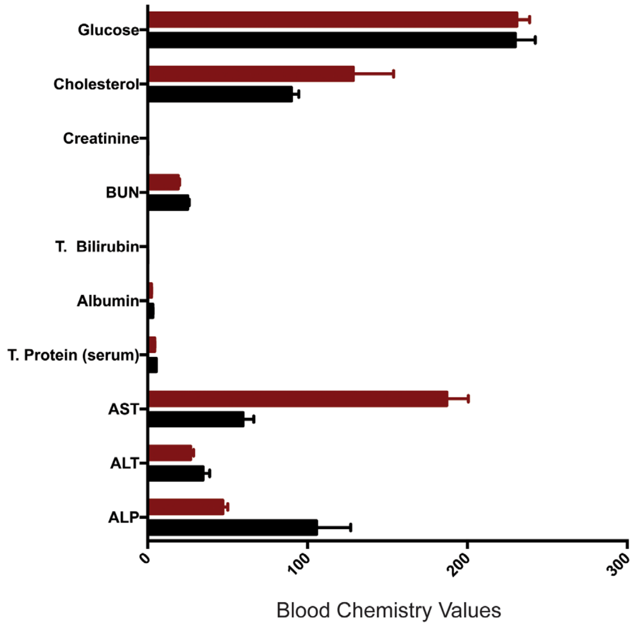

Reference Clinic Chemistry Values

	Unit	Mouse
Glucose	mg/dl	62 - 175
Cholesterol	mg/dl	-
Creatinine	mg/dl	0.2 - 0.9
BUN	mg/dl	8 - 33
T. Bilirubin	mg/dl	0 - 0.9
Albumin	g/dl	2.5 - 3
Total Protein(serum)	g/dl	3.5 - 7.2
AST	U/L	54 - 298
ALT	U/L	17 - 77
ALP	U/L	35 - 96

B

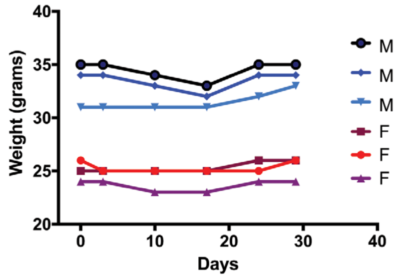

C

Urinalysis

Day	pH	Leukocytes	Protein	Glucose	Blood
3-5	6	Trace	Trace	Normal	-
10-11	5	-	Trace	Normal	-
18-22	5	Trace	Trace	Normal	-
26	6	Trace	Trace	Normal	-

D

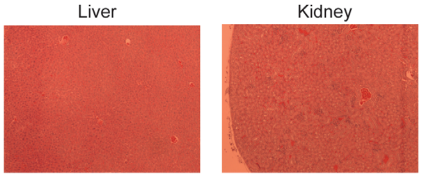

Supplementary Figure 6: Liver and kidney toxicities of CT20p-NPs. Three male and three female nude mice (6-8 weeks old) were treated with five doses (2.4 mg/kg, 4.8 mg/kg, 9.6 mg/kg, 19.2 mg/kg, 38.4 mg/kg) of CT20p-NPs over a two-week period. Treatments were performed on Days 0, 3, 7, 10 and 14 and mice were euthanized 14 days after the last treatment. **(A)** Serum of treated mice was sent to Idexx for testing for the chemical chemistry panel which assesses kidney and liver function. No statistical differences were observed between control and NP-treated mice. Reference table contains normal clinical chemistry values adapted from the Animal Care Website at the University of Arizona. **(B)** The weight of mice during treatment was determined at the specified time points. **(C)** Urine analysis was performed during treatment at four time points as indicated in the table. Results from a representative mouse are shown. **(D)** Histology of treated mice liver and kidney. Representative images are shown.

Supplementary Table 1: Sample sizes for lung tissue cores analysis used in Figure 1

	Classification	Sample size
Lung	Normal	20
	Adenocarcinoma	76
	Carcinoid	11
	Small cell carcinoma	82
	Squamous cell carcinoma	67
	SqCLC T1/T2	52
	SqCLC T3/T4	15
	SCLC T1/T2	67
	SCLC T3/T4	15

The numbers indicated correspond to the total numbers of tissue cores that received a score. Specific TMA reference numbers can be found in Materials and Methods.

Supplementary Table 2: Sample sizes for liver, colon and prostate tissue cores analysis used in Supplementary Figure 2

	Classification	Sample size
Liver	Normal hepatic tissue	20
	Cholangiocellular carcinoma	30
	Hepatocellular carcinoma	147
	HCC - grade 1	19
	HCC - grade 2	111
	HCC - grade 3	12
	**Classification**	**Sample size**
Colon	Normal colon tissue	7
	Adenocarcinoma	23
	Mucinous adenocarcinoma	8
	Signet cell ring carcinoma	7
	**Classification**	**Sample size**
Prostate	Normal prostate tissue	9
	Adenocarcinoma	131
	Adenocarcinoma - stage II	33
	Adenocarcinoma - stage III	10
	Adenocarcinoma - stage IV	27

The following TMAs were used for analysis shown in Supplementary Figure 1: BC03118 (hepatocellular carcinoma), PR803b and PR631 (prostate carcinoma), CO484a (colonic carcinoma). Table indicates number of cases per analysis shown in graphs S1A, S1E and S1I.

Supplementary Table 3: Cell lines origin, source, mutations and lot number

	Cell line	Tissue	Source	Key characteristics/ mutations	Lot number
**Small Cell Lung Cancer**	NCIH1882 (ATCC CRL5903)	Lung	Metastatic site:Bone marrow	Adherent cell line/ RB1 mutation	58136167
	NCIH1048 (ATCC CRL5853)	Lung	Metastatic site: Pleural effusion	Adherent cell line - cells grow in colony and can form clusters/ RB1, TP53 mutations	62784665
	NCIH1417 (ATCC CRL5869)	Lung	Primary	Suspension cells - grow in floating clusters/ RB1, TP53 mutations	62007515
	NCIH1105 (ATCC CRL5856)	Lung	Metastatic site: Lymph node	Suspension cells - grow in floating clusters/ TP53 mutations	63374473
	NCIH719 (ATCC CRL5837)	Lung	Metastatic site:Bone marrow	Suspension cells - grow in floating clusters/ RB1, TP53 mutations	62007616
**Normal Tissue Immortalized**	THLE2 (ATCC CRL2706)	Liver	Liver	Epithelial cells transformed with SV40 large T antigen [1].	64258509
	MCF-10A (ATCC CRL-10317)	Breast	Breast epithelial	Breast epithelial cells, derived from human fibrocystic mammary tissue spontaneously immortalized [2].	64066742
	AC16 (SCC109)	Heart	Cardiomyocyte	Primary cells from human ventricular tissue, were fused with SV40 transformed, uridine auxotroph human fibroblasts, devoid of mitochondrial DNA [3].	RD1606008

Table summarizes information pertaining to cell lines used in the experiments in the main manuscript. Information about tissue of origin, source (primary versus metastatic sites for tumor derived cell lines), key characteristics and lot number are provided. Relevant publications regarding the immortalized cell lines used are provided.

Supplementary Table 4: Comparison of CCT2 levels, CT20p susceptibility, tumor source, and key genes mutated

	CCT2 Levels	CT20p susceptibility	Source	Culture type	Mutations
NCIH1048 (ATCC CRL5853)	↑↑↑	↑↑↑	Metastatic site: Pleural effusion	Adherent	RB1, TP53
NCIH1417 (ATCC CRL5869)	↑↑↑	↑	Primary	Suspension	RB1, TP53
NCIH1105 (ATCC CRL5856)	↑↑↑	↑↑	Metastatic site: Lymph node	Suspension	TP53
NCIH1882 (ATCC CRL5903)	↑↑	↑	Metastatic site:Bone marrow	Adherent	RB1, TP53
NCIH719 (ATCC CRL5837)	↑	↑	Metastatic site:Bone marrow	Suspension	RB1, TP53

	CCT2 levels: based on normalized levels (Fig.2E)	CT20p susceptibility
	Normalized CCT2 levels making levels obtained for H1048=100%	Extrapolated IC50 concentrations based on results otained from the two doses tested (linear regression)
↑↑↑	> 70%	< 75 µg/mL
↑↑	< 69% and > 30%	> 76 µg/mL and < 120 µg/mL
↑	< 30%	> 121 µg/mL
	% of signal	µg/mL of nanoparticle

This table summarizes results on experiments using the five SCLC cell lines obtained from CCT2 blots found in Figure 2, CT20p susceptibility found in Figure 3, tumor source, culture type (suspension versus adherent) and key genes containing mutations. The criteria for "high, medium and low levels" are summarized on the table itself. In short, for CCT2 levels, Figure 2E was used. Cell lines with signal about 70% were classified as "high", and below 30% as low, if in between, they were considered "medium". For CT20p susceptibility, average values obtained for untreated, 75µg/ml and 150µg/ml were plotted in prism and using linear regression, the IC50 value for each cell line was determined. If the extrapolated IC50 was below 75µg/ml, susceptibility was considered high, if above 121µg/ml, susceptibility was determined to be low, if in between, cells were considered partially susceptible to CT20p treatment.

Supplementary Table 5: Sample size for CCT2/STAT3 staining intensity correlation used in Figure 4A

US. Biomax reference number	Sample size
**LC802c**	**80**

SCLC		Normal	Adjacent
**70**		**6**	**4**
# equal scores	# of scores +/- 1	not used in the analysis	
49	20		
Total = 69 cores			
**Core C9 Staining was inconclusive, therefore it was not included in the analysis**			

The numbers indicated correspond to the total numbers of tissue cores that received a score.
